# Spatially and functionally distinct subclasses of breast cancer-associated fibroblasts revealed by single cell RNA sequencing

**DOI:** 10.1038/s41467-018-07582-3

**Published:** 2018-12-04

**Authors:** Michael Bartoschek, Nikolay Oskolkov, Matteo Bocci, John Lövrot, Christer Larsson, Mikael Sommarin, Chris D. Madsen, David Lindgren, Gyula Pekar, Göran Karlsson, Markus Ringnér, Jonas Bergh, Åsa Björklund, Kristian Pietras

**Affiliations:** 1 Division of Translational Cancer Research, Department of Laboratory Medicine, BioCARE, Lund University, Medicon Village, 22381 Lund, Sweden; 2 Department of Biology, National Bioinformatics Infrastructure Sweden, Science for Life Laboratory, Lund University, Sölvegatan 35, 22362 Lund, Sweden; 3 Department of Oncology and Pathology, Karolinska Institutet, Karolinska Universititetssjukhuset Z1:01, 17176 Stockholm, Sweden; 4 Division of Molecular Hematology, Lund Stem Cell Center, Lund University, BMC B12, 22184 Lund, Sweden; 5 Division of Oncology and Pathology, Department of Clinical Sciences, Lund University, Skåne University Hospital, 22185 Lund, Sweden; 6 Department of Cell and Molecular Biology, National Bioinformatics Infrastructure Sweden, Science for Life Laboratory, Uppsala University, Husargatan 3, 75237 Uppsala, Sweden

## Abstract

Cancer-associated fibroblasts (CAFs) are a major constituent of the tumor microenvironment, although their origin and roles in shaping disease initiation, progression and treatment response remain unclear due to significant heterogeneity. Here, following a negative selection strategy combined with single-cell RNA sequencing of 768 transcriptomes of mesenchymal cells from a genetically engineered mouse model of breast cancer, we define three distinct subpopulations of CAFs. Validation at the transcriptional and protein level in several experimental models of cancer and human tumors reveal spatial separation of the CAF subclasses attributable to different origins, including the peri-vascular niche, the mammary fat pad and the transformed epithelium. Gene profiles for each CAF subtype correlate to distinctive functional programs and hold independent prognostic capability in clinical cohorts by association to metastatic disease. In conclusion, the improved resolution of the widely defined CAF population opens the possibility for biomarker-driven development of drugs for precision targeting of CAFs.

## Abstract (web summary)

Cancer-associated fibroblasts (CAFs) are an important component of the breast tumour microenvironment. Here, single-cell RNA sequencing of CAFs from a mouse model of breast cancer defines three transcriptomically distinct subpopulations with putatively different functions.

## Introduction

The traditional tumor cell-centric view of cancer has been revised during the past decades with the increasing appreciation of the importance of the tumor microenvironment for the malignant phenotype. The elucidation of reciprocal interactions of cancer cells with their local milieu has inspired the development of conceptually novel targeted therapeutics with the aim to thwart paracrine signaling between different cell types of the tumor mass. The cancer-associated fibroblast (CAF) comprises the most prevalent constituent cell type in the tumor microenvironment in many cancers, including breast, pancreas, and hepatic carcinomas^1,2^ and has been documented to endorse many, if not all, hallmarks of cancer^3^. Cell morphology is still the most reliable way to distinguish CAFs within the tumor parenchyme, as commonly used cellular markers, such as α-smooth muscle actin (SMA), fibroblast-specific protein 1 (FSP-1/S100A4), or fibroblast activation protein (FAP) are neither all-encompassing nor completely specific. The lack of congruency in marker expression raises the possibility that CAFs comprise a diverse group of cells made up of several subtypes^4^. Support for this notion comes from recent studies of e.g., pancreatic ductal adenocarcinoma^5^, breast carcinoma^6–8^, colon carcinoma^9^, and lung adenocarcinoma^10^, in which functionally distinct subclasses of CAFs were identified by various means based on expression of a limited set of markers. In addition, CAFs have been suggested to originate from various sources, including resident fibroblasts, bone marrow-derived mesenchymal stem cells, pericytes, and malignant cells or endothelial cells that have undergone a mesenchymal transition^11,12^, further indicating a diversity within the fibroblast population.

Single-cell RNA-sequencing (scRNA-seq) is a technological innovation that overcomes the masking of cellular subsets within the data from bulk RNA sequencing and allows investigation of the transcriptome of individual cells with the aim to define subpopulations of cells inferred by similar transcriptional programs. In tumors, transcriptome analysis of single cells derived from melanoma patients clearly defined clusters of malignant and non-malignant cell types, shedding light on the interaction of stromal and immune cells in the context of tumor growth^13^. Similarly, a recent analysis of colorectal cancers employing scRNA-seq categorized cells into constituent cell types, including CAFs, based on marker expression^9^. Also, scRNAseq has been utilized to identify CAFs as a specific responder population to stimulation with Hedgehog, which in turn will instigate a CAF-induced cancer stem cell niche^7^. However, previous studies have not been designed to specifically dissect a broadly defined cell type within the tumor, such as CAFs, into distinct cellular subsets due to restrictions in the number of cells analyzed and limitations in the scRNA-seq methodology.

Here, we use the highly sensitive Smart-seq2 protocol to delineate the heterogeneity of 768 CAFs isolated from the genetically engineered MMTV-PyMT mouse model of breast cancer^14,15^. We define three transcriptionally diverse subpopulations of CAFs. Notably, each CAF subset is clearly discriminated by the expression of gene programs representing different functionality and is demonstrated to have a unique spatial location within the tumor parenchyme. Thus, our work dissects the CAF population within breast tumors at single cell resolution and reveals a previously unappreciated functional diversity within the tumor microenvironment that opens up for further development of tools for precision medicine.

## Results

### Single cell RNA-seq reveals subpopulations of breast CAFs

**Fig. 1:**
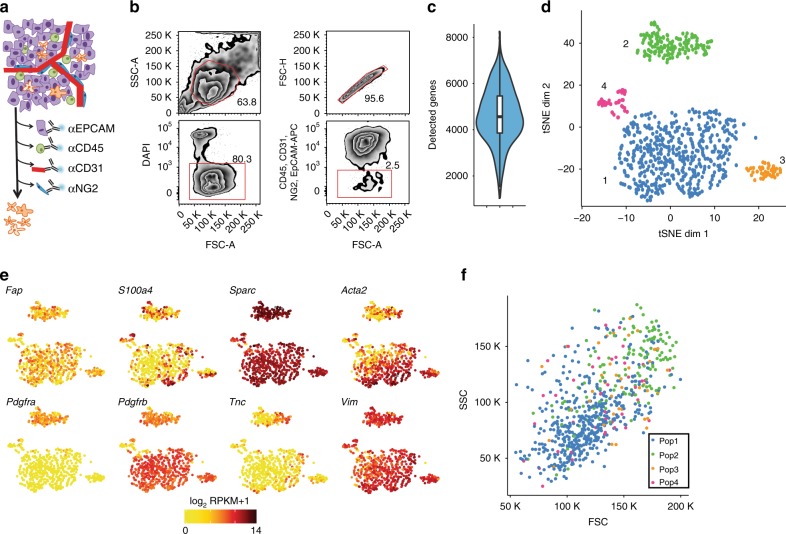
Unbiased clustering of fibroblast single cell transcriptomic data reveals four populations. **a** Schematic representation of negative selection strategy removing CD31^+^, CD45^+^, NG2^+^, and EPCAM^+^ cells. **b** Gating strategy and quantification of flow cytometry for single cell sequencing. After gating out doublets and DAPI^+^ dead cells, EpCAM^−^CD31^−^CD45^−^NG2^−^ CAFs made up 2.5% of the cells. FSC forward scatter, SSC side scatter. For single marker staining see also Supplementary Figure 1. **c** Violin plot of detected genes in 784 sorted fibroblasts. **d** *t*-SNE layout of CAFs (*n* = 716) by RPKM-normalized transcriptomic data. Colors represent clusters assigned by DBSCAN. **e** Expression plots on *t*-SNE layout. log_2_(RPKM + 1) levels of CAF marker genes in individual cells. **f** Cell size and granularity as determined by forward-scattered light (FSC) and side-scattered light (SSC) of different CAF populations

To improve the taxonomy of CAFs in breast cancer at the cellular and functional level, we performed scRNA-seq on isolated mesenchymal cells from tumors of the MMTV-PyMT mouse model of breast cancer. Due to the lack of a common CAF marker, and due to the prospect to uncover previously unknown subsets of CAFs, we used a negative selection fluorescence-activated cell sorting (FACS) strategy to isolate an EpCAM^−^/CD45^−^/CD31^−^/NG2^−^ cell fraction devoid of epithelial cells, immune cells, endothelial cells, and pericytes, respectively (Fig. 1a, b and Supplementary Figure 1a-e). The isolated fraction comprised 2.5% of the single viable cells derived from late carcinomas of 14 weeks old MMTV-PyMT mice. Immunostaining of cytospins of the isolated cells for the CAF markers PDGFRα and α-SMA confirmed the purity of the obtained population, as most cells stained positively for one or both markers (Supplementary Figure 1f). Libraries for scRNA-seq were prepared in two 384-well plates harboring CAFs from one tumor each, and sequenced using the Smart-seq2 protocol with exogenous RNA controls from External RNA Controls Consortium (ERCC) spiked into the cell lysates^16^.

Based on five quality control metrics, 52 out of 768 libraries were filtered out due to low quality (Supplementary Figure 1g-k). Genes with fewer than one count on average over all cells were removed, resulting in a final of 10,835 endogenous genes and 53 spike-ins kept for further downstream analysis. Each individual cell contained transcripts of an average of approximately 4600 distinct genes (Fig. 1c). To investigate whether the isolated pool of CAFs represented different subclasses of cells, we performed dimensionality reduction by different methods. Indeed, principal-component analysis (PCA) of the expression of the 557 genes with the highest biological variation as determined in comparison to ERCC spike-ins (Supplementary Figure 2a) resulted in two main clusters and one small cluster in a scatterplot of the first two principal components (Supplementary Figure 2b). Further, based on the same gene set, two-dimensional projection by *t*-distributed stochastic neighbor embedding (*t*-SNE) grouped the cells distinctly into four groups identified by DBSCAN, designated Population 1–4, demonstrating the existence of subtypes of CAFs with discrete gene expression profiles (Fig. 1d). Since cells from both tumors clustered in a similar way with both PCA and *t*-SNE, and in general exhibited similar quality metrics, we continued the analysis without taking the origin of the cells into further consideration (Supplementary Figure 2b, c). Importantly, the negative selection markers *Epcam*, *Pecam1*, and *Ptprc* were not appreciably detected in any cell, excluding the possibility that the observed cell clusters resulted from analyzing a mixture of mesenchymal and non-mesenchymal cell types (Supplementary Figure 2d-f). However, modest levels of transcript from the *Cspg4* gene encoding NG2 were detected in Populations 1 and 3, despite selecting against cells with NG2 protein expression (Supplementary Figure 2g), suggesting either negligible surface exposure of the NG2 protein or post-transcriptional regulation of the mRNA.

### Subpopulations of CAFs harbor distinct gene programs

Next, we explored the expression of prototypical CAF markers within the cellular subtypes. Although we detected expression of at least one CAF marker in every cell, only the non-specific mesenchymal marker transcripts *Vim* and *Sparc* were expressed by most cells, highlighting the need to better delineate both general and distinctive molecular features of the CAF populations (Fig. 1e). Notably, *Pdgfra* was specifically expressed by cells in Population 2, whereas *Pdgfrb* was expressed by all cells apart from Population 4. *Fap*, *S100a4* (encoding FSP-1) and *Acta2* (encoding α-SMA) displayed a salt-and-pepper expression pattern in all four CAF populations. In addition, the two major CAF subtypes, i.e., Population 1 and Population 2, also differed in cell size, as indicated by the FACS data, further suggesting that the cellular subgroups represented entities with separate biophysical properties (Fig. 1f). In order to confirm the clustering, we made use of the SC3 R package developed for single-cell transcriptomics^17^ and obtained a similar clustering as previously observed by *t*-SNE (Supplementary Figure 3a).

Since production and modification of the extracellular matrix (ECM) are key functions of fibroblasts, we investigated the transcription of genes encoding ECM proteins included in the matrisome^18^ to seek biological validation of the CAF subpopulations. Indeed, based on unsupervised hierarchical clustering of the matrisome gene set, we observed that the 716 cells clustered according to our previously defined CAF populations, with the exception that Populations 1 and 3 were intermingled with each other (Supplementary Figure 3b). All populations displayed a unique expression signature of matrisome genes, supporting the notion that each of the CAF populations produced a distinct matrix with a specific biological function. Population 2 harbored the strongest ECM signature with a generally high expression of matrisome genes.

**Fig. 2:**
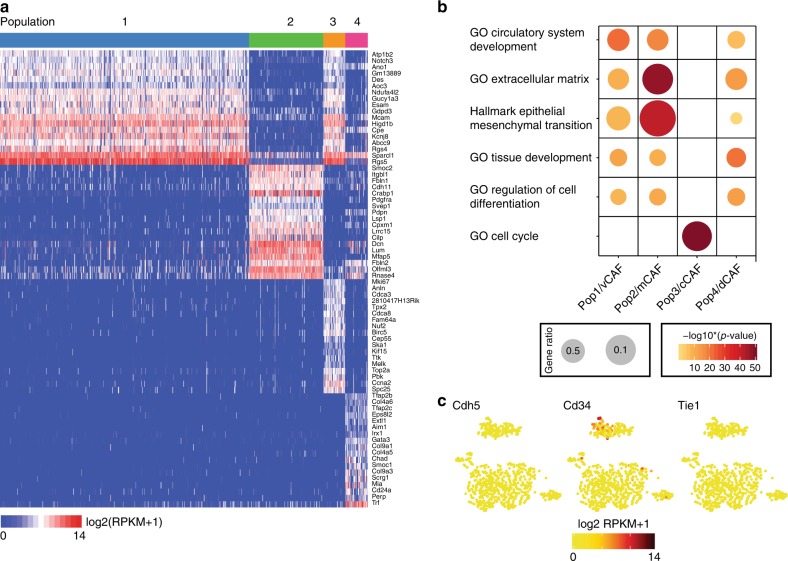
Distinct gene profiles in CAF subpopulations account for functional differences. **a** Heatmap of top 18 differentially expressed genes in each subpopulation estimated by ROTS. **b** Enrichment of the 150 most SDE in gene ontology (GO) terms. Gene ratio is determined by the number of detected genes within a GO term compared to the total number of genes. **c** Expression plots based on *t*-SNE layout. log_2_(RPKM + 1) levels of endothelial cell genes in individual cells

In order to detect differentially expressed genes that specifically distinguished each CAF subtype, we performed reproducibility-optimized test statistic (ROTS)^19^ for the defined populations. Each population was compared to the other pooled populations to find unique gene signatures and upregulated genes with an FDR <0.001 were considered significantly differentially expressed (SDE). We detected 1999 SDE genes in Population 2, whereas Populations 1, 3, and 4 harbored 522, 590, and 859 SDE genes, respectively. The top 18 SDE genes of each population are represented in the heatmap depicted in Fig. 2a. We confirmed the result of the ROTS function by applying commonly used algorithms, such as SCDE^20^, edgeR^21^, DESeq2^22^, and Wilcoxon rank-sum test^23^, obtaining closely overlapping lists of SDE genes (Supplementary Figure 4a).

Next, we used the first 150 SDE genes of each population to define gene signatures by gene ontology (GO) that functionally described each subpopulation (Fig. 2b). The SDE genes of Population 1 were significantly enriched for GO sets for vascular development and angiogenesis (Fig. 2b), and we therefore termed this subtype vascular CAFs (vCAFs). Population 2 SDE genes were enriched for GO sets related to the ECM and EMT (of note, this gene set contains mainly matrix-related genes), confirming our previous hierarchical clustering using the matrisome gene set (Fig. 2b and Supplementary Figure 3b). Due to the strong ECM signature, cells in Population 2 were named matrix CAFs (mCAFs). Cell cycle-related gene sets dominated the SDE genes from Population 3 (Fig. 2b). In agreement with the GO classification, the trained cell cycle classifier Cyclone^24^ identified the majority of cells in Population 3 to be in the G2, M, or S phase of the cell cycle, whereas cells in other clusters were predominantly classified to be in the G1 phase of the cell cycle (Supplementary Figure 4b). Consequently, Population 3 cells were termed cycling CAFs (cCAFs). Based on the SDE genes, gene sets detected for Population 4 were connected to differentiation of cells, as well as the development and morphogenesis of tissues (Fig. 2b); we thus labeled this subtype as developmental CAFs (dCAFs).

### vCAFs originate from a perivascular location

**Fig. 3:**
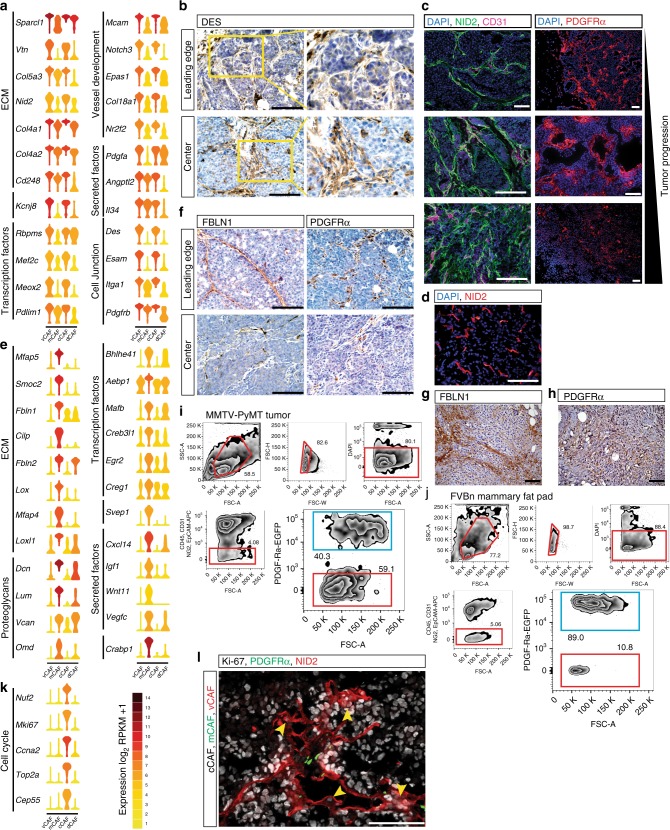
vCAF and mCAF marker genes can be used to trace back subpopulations in tissue sections. **a** Violin plots of selected vCAF differentially expressed genes in log_2_(RPKM + 1). Violin colors represent mean expression of each population. Genes were sorted based on gene ontology terms. **b** Immunohistochemistry (IHC) staining of desmin on MMTV-PyMT tumor sections (6 µm). Images were acquired from the leading edge and the tumor center. Yellow boxes (left) indicate 2× magnified area (right). **c** IF staining of Nidogen-2 (green) and CD31 (magenta) or PDGFRα (red) on MMTV-PyMT tumor sections (5 µm) from mice of age 8 weeks, 12 weeks, and 15 weeks (top to bottom). Nuclei were counterstained with DAPI. **d** Immunofluorescence (IF) staining of Nidogen-2 on human tumor tissue (5 µm). Nuclei were counterstained with DAPI. **e** Violin plots of selected mCAF differentially expressed genes in log_2_(RPKM + 1). **f** IHC staining of fibulin-1 and PDGFRα in MMTV-PyMT tumor sections (6 µm). Images were acquired from the leading edge and the tumor center. IHC staining of fibulin-1 (**g**) and PDGFRα (**h**) in human tumor tissue sections (6 µm). FACS-sort of MMTV-PyMT tumor (**i**) and mammary gland (**j**) tissue. Gating on single, living CD45^−^CD31^−^NG2^−^EPCAM^−^ cells followed by gating on PDGFRα^+^ cells (blue box) or PDGFRα^-^ cells (red box). **k** Violin plots of cell cycle gene expression in log_2_(RPKM + 1). **l** IF staining for Nidogen-2 (red) and Ki-67 (grey) on sections (5 µm) from PDGFRα-EGFP (green) reporter mice. Arrows indicate Nid2^+^Ki67^+^ cCAF. Scale bar: 50 µm

Due to the close correlation of the SDE genes in vCAFs with genes involved in vascular development, we investigated the expression of prototypical marker genes for endothelial cells, such as *Cdh5*, *Pecam1*, *Cd34*, and *Tie1* to rule out inadvertent contamination of the vCAF population. Reassuringly, we did not find evidence for meaningful expression of endothelial cell markers in any of our analyzed cells (Fig. 2c). Instead, the SDE genes for vCAFs included vascular regulators such as *Notch3*, *Epas1*, *Col18a1*, and *Nr2f2* (Fig. 3a). In addition to genes controlling angiogenesis, transcription factors and genes involved in cell junctions were also prominently represented in the specific transcriptomes of vCAFs (Fig. 3a). We confirmed the high abundance of vCAFs within the stromal compartment of tumors from MMTV-PyMT mice using desmin as a marker (Fig. 3b). Notably, the proportion of mesenchymal stromal cells positive for vCAF markers was distinctly higher in the tumor core, compared to the leading edge of the tumor (Fig. 3b). In accordance with their apparent function, vCAFs predominantly localized in proximity to the vasculature, as shown by immunostaining for the vCAF marker Nidogen-2 and the endothelial cell marker CD31 (Fig. 3c). Strikingly, we observed Nidogen-2-positive cells to be tightly associated with blood vessels in early stages of tumor development (8 weeks old MMTV-PyMT mice). During the course of tumor progression, increasing amounts of Nidogen-2-positive cells were found detached from vessels, showing streaks of cells infiltrating the stroma of tumors from 12- and 15-weeks-old mice. Since Nidogen-2 is a secreted protein, we confirmed its validity as a vCAF marker in immunostainings by combining RNAscope in situ hybridization (RNA-ISH) for vCAF transcripts *Kcnj8* and *Notch3* with immunostaining of Nidogen-2 (Supplementary Figure 4c). Gratifyingly, immunostaining of Nidogen-2 in human breast tissues showed a stromal expression pattern (Fig. 3d) and a similar pattern was observed in human breast carcinomas included in The Human Protein Atlas (http://www.proteinatlas.org)^25^, providing independent evidence (Supplementary Figure 4d). We furthermore detected Nidogen-2-positive stromal cells in tumors from orthotopic transplantation models, including the murine cell lines 4T1 and EO771 as well as the human breast cancer cell line MDA-MB-231 (Supplementary Figure 4e). Thus, based on their histological localization, we conclude that the vCAF subclass originates from a pool of perivascular cells that later invades the tumor stroma over the course of tumor progression.

### mCAFs are descendants of resident fibroblasts

The mCAF subset of the tumor stroma specifically expressed transcripts of a large variety of ECM-related genes, such as glycoproteins (*Dcn*, *Lum*, and *Vcan*), structural proteins (*Col14a1*), matricellular proteins (*Fbln1*, *Fbln2*, and *Smoc*), and matrix-modifying enzymes (*Lox* and *Loxl1*) (Fig. 3e). Additionally, mCAFs abundantly expressed the immune cell-attracting factor *CXCL14*, suggestive of a role in the regulation of the tumor immune response. Immunostaining of the mCAF markers Fibulin-1 and PDGFRα showed high prevalence of positive cells at the invasive front of tumors, in contrast to the relatively low abundance of mCAFs in the tumor core (Fig. 3f). The two mCAF markers Fibulin-1 and PDGFRα identified a profuse infiltration of mCAFs in the tumor stroma of human breast cancer tissue (Fig. 3g, h and Supplementary Figure 4d,f). In contrast to vCAFs, the relative number of mCAFs decreased during tumor progression in the MMTV-PyMT mouse model (Fig. 3c). In orthotopic grafting models we observed mCAFs mainly in the syngrafts, but only sparsely in the xenograft model (Supplementary Figure 4e). Intriguingly, and in contrast to the malignant tissue from 12 weeks old and 14 weeks old mice where mCAFs represented 40.3% and 20.0% of the total CAF pool, respectively, 89.0% of fibroblasts isolated from the mammary gland of non-transgenic FVB/N mice expressed mCAF markers, as detected by FACS (Figs. 1d and 3i, j). Based on the similar marker expression of mCAFs and the dominant fibroblast population in the normal mammary gland, we conclude that mCAFs may derive from resident fibroblasts that are co-opted by the tumor.

### cCAFs are the proliferating segment of vCAFs

SC3 clustering using the matrisome gene set (Supplementary Figure 3b) demonstrated that cCAFs and vCAFs clustered together. Indeed, only cell cycle genes were found to be differentially expressed between cCAFs and vCAFs (Fig. 3k). Furthermore, immunostaining for the proliferation marker Ki-67 demonstrated that dividing stromal fibroblasts were predominantly found within nests of vCAFs, and not mCAFs, thus localizing cCAFs in situ and strengthening the proposition that cCAFs are indeed vCAFs currently engaged in cell division (Fig. 3l). Therefore, we conclude that cCAFs represent the proliferative segment of vCAFs; based on their relative abundance at the time of isolation, 7.7% of the cells within the vCAF population were dividing.

### dCAFs share expression patterns with the tumor epithelium

**Fig. 4:**
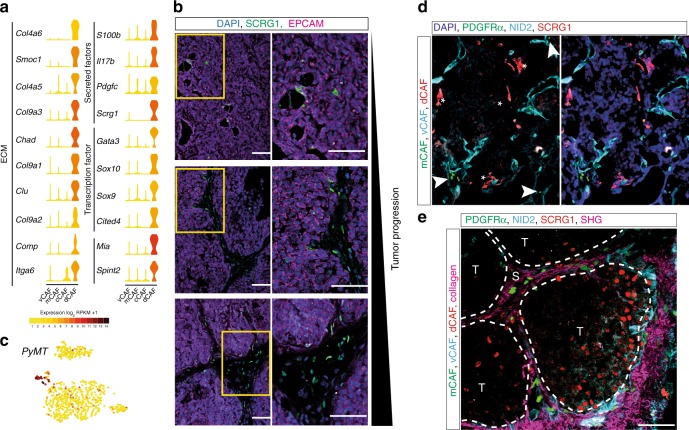
dCAF express the oncogenic driver gene and share gene expression with the tumor epithelium. **a** Violin plots of selected dCAF differentially expressed genes in log_2_(RPKM + 1). Violin colors represent mean expression of each population. Genes were sorted based on gene ontology terms. **b** IF staining of SCRG1 (green) and EPCAM (magenta) on MMTV-PyMT tumor sections (5 µm) from mice of age 8 weeks, 12 weeks, and 15 weeks (left to right). Nuclei were counter stained with DAPI (blue). Yellow boxes indicate the area magnified in the lower panel. **c** Expression plots based on *t*-SNE clustering. log_2_(RPKM + 1) levels of the virus-derived oncogenic driver gene in individual cells. **d** IF staining of Nidogen-2 (cyan) and SCRG1 (red) on MMTV-PyMT tumor tissue derived from PFGFRα-EGFP (green) reporter mice. Nuclei are stained with DAPI (blue). White arrowheads and stars indicate mCAF and dCAF, respectively. **e** IF staining of Nidogen-2 (cyan) and SCRG1 (red) on MMTV-PyMT tumor tissue derived from PFGFRα-EGFP (green) reporter mice. The image was acquired by 2-photon microscopy. Collagen fibers were detected by second harmonic generation (SHG, magenta). Dotted lines separate malignant tissue (T) from stroma (S). Scale bar: 50 µm

Apart from harboring a distinct profile of ECM genes, dCAFs were distinguished by the expression of genes related to various kinds of stem cells (*Scrg1*, *Sox9*, and *Sox10*, among others) (Fig. 4a), in keeping with their putative function in tissue development. dCAFs, as identified by the specific marker SCRG1, were scarce in tumor tissue from MMTV-PyMT mice, in agreement with the low number of cells from this subtype that were isolated from the original tumors (Figs. 1d and 4b). Interestingly, dCAFs were intermingled with the malignant epithelium during early stages of tumor development, whereas SCRG1-positive cells could be found both within the epithelium and in stromal streaks of late-stage tumors (Fig. 4b). SCRG1 expression could also be detected in human tissue in a similar distribution (Supplementary Figure 4d). Intriguingly, expression of the transgenic PyMT oncogene was strongly detected in dCAFs, indicating a malignant cell origin for this subset of cells (Fig. 4c).

We next used RNA-ISH in order to detect dCAF-specific transcripts in tumor tissue with a variety of markers (Supplementary Figure 5a,b). The *Mia* transcript was mainly detected at low levels in the tumor epithelium, with a few sparse hotspots of increased expression (Supplementary Figure 5a). The *Spint2* transcript was homogenously expressed in the tumor epithelium, but not in the stroma, as identified by the mCAF-specific transcript *Mfap5* (Supplementary Figure 5b). Immunostaining of human tissues confirmed MIA expression in the tumor epithelium with few discrete MIA-positive CAFs within stromal streaks (Supplementary Figure 5c and 4f). Taken together, the overlapping expression of dCAF SDE genes in both tumor epithelium and in stromal mesenchymal cells suggests that dCAFs may originate from tumor cells that have undergone an epithelial-to-mesenchymal transition (EMT).

### CAF subclasses represent histologically distinct entities

In order to conclusively demonstrate the existence of spatially distinct subsets of CAFs within malignant lesions, we visualized all subsets by using fluorescent reporters or immunostaining. Indeed, simultaneous detection of the vCAF marker Nidogen-2, the mCAF marker PDGFRα, and the dCAF marker SCRG1, identified three distinct stromal populations with divergent growth patterns and localization in relation to the nests of tumor cells (Fig. 4d). To obtain a better representation of the distribution of CAF subpopulations and other constituent components of the tumor, we made use of 2-photon confocal microscopy. The resulting images again revealed three distinct populations of CAFs defined by the specific markers (Fig. 4e). Importantly, PDGFRα-positive mCAFs were found to reside predominantly within collagen-rich streaks, in keeping with their inferred role as providers of ECM. Additionally, SCRG1-positive dCAFs were located on the tumor-stroma boundary, suggestive of their putative origin as malignant epithelial cells. Finally, the Nidogen-2-positive vCAFs were distributed along vessels, as well as in stromal streaks.

To confirm that the detected CAF subpopulations were distinct using more markers, we used RNA-ISH of vCAF and mCAF marker transcripts and observed no overlap in the expression of several pairs of markers (Supplementary Figure 5d-e). In contrast, the expected partial overlap was detected between the expression of the mCAF marker *Svep1* and the commonly used, but promiscuous CAF marker *Acta2* (Supplementary Figure 5f). Finally, we confirmed the mutual-exclusivity of mCAF and vCAF marker genes Fibulin-1 and Nidogen-2 in human tissue sections by immunostaining (Supplementary Figure 5g).

### Subpopulations of CAFs are independent prognostic biomarkers

**Fig. 5:**
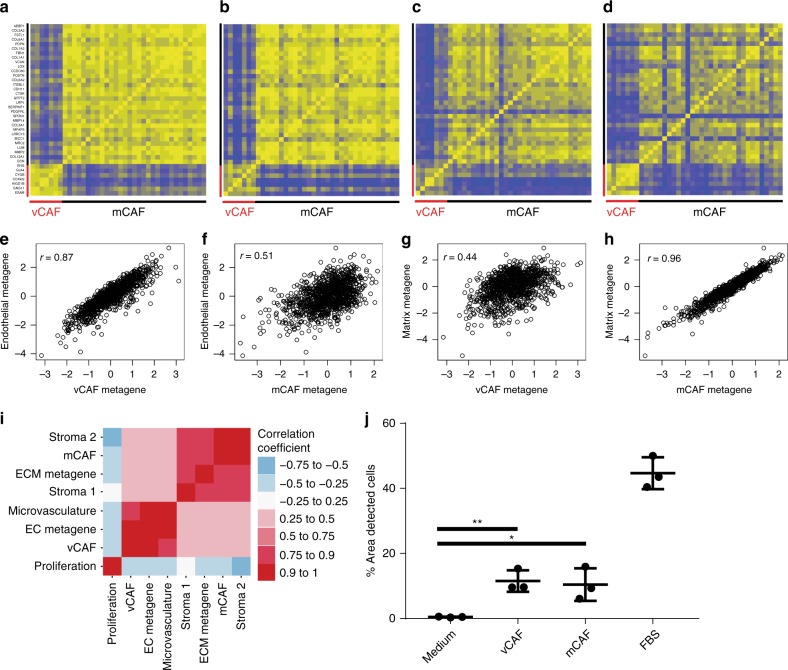
CAF gene profiles correlate in human bulk RNA-sequencing data. Pearson correlation of genes from vCAF and mCAF profiles in TCGA datasets of **a** breast cancer, **b** pancreatic ductal adenocarcinoma, **c** lung adenocarcinoma, and **d** renal clear cell carcinoma. Correlation of the vCAF profile to **e** an endothelial metagene and **f** a matrix metagene. Correlation of the mCAF profile to **g** an endothelial metagene and **h** a matrix metagene in TCGA breast cancer data. **i** Correlation of vCAF and mCAF profiles to functional metagenes in the nested case–control study dataset of breast cancer patients. **j** Quantification of transwell invasion assay. The average percentage of area covered by invaded cells on the bottom of the membrane was quantified from 4 representative images in *n* = 3 biological repeats; Data depicted as mean ± s.d. *P = 0.026, **P = 0.0045; two-sided, unpaired Students *t*-test

We next set out to determine whether the observed CAF subtypes could be identified in bulk RNA-seq data from human patient samples. We reasoned that subclasses of cells would be best detected by using distinguishing gene expression profiles consisting of highly correlated genes, as co-regulation of transcriptional programs in bulk data would be an indicator of a common cellular origin. Thus, using bulk RNA-seq data from The Cancer Genome Atlas (TCGA) database for breast cancer^26^, we identified highly correlated genes among the SDE genes from each CAF subtype, resulting in a condensed profile of 7 genes for vCAFs and 30 genes for mCAFs (Supplementary Table 1). The profiles were specific for each cellular subset compared to the others, thus further indicating the existence of CAF subtypes also in human breast tumors (Fig. 5a). In addition, the gene profiles were also highly and specifically correlated within bulk RNA-seq data from other cancers, such as pancreatic adenocarcinoma, lung cancer, and renal cell cancer, suggestive of a certain extent of commonality in the development of CAFs within distinct malignant diseases (Fig. 5b–d). When using the same approach for dCAFs, only 2 genes remained with a correlation coefficient >0.7, indicating that many of the genes that signify dCAFs compared to other CAF populations may also be expressed by other cell types within the tumor tissue, or alternatively that dCAFs are very scarce, in keeping with their putative origin from a transient EMT (Supplementary Table 1). We did not attempt to derive a gene profile for cCAFs, since the strategy would be irrevocably confounded by the overarching expression of proliferation-related genes.

Next, in order to determine whether the functionally distinct gene programs of vCAFs and mCAFs could also be discerned from the analysis of bulk data from the TCGA database, we investigated the correlation between the condensed gene profiles for each cellular subtype and metagenes for their inferred functions, i.e., regulation of angiogenesis and ECM production. In keeping with the data from mouse tumors, the vCAF signature was highly correlated to an endothelial cell metagene in breast tumors, whereas the mCAF signature was strongly associated with an ECM metagene^27^ (Fig. 5e–h). Notably, the converse relations were not observed, strongly indicating conservation of the functionally distinct gene programs of vCAFs and mCAFs between mouse and human tumors. Also, similar findings were made using transcriptional data from pancreatic adenocarcinoma (Supplementary Figure 6a).

CAFs have been suggested to be important regulators of crucial parameters for determining the prognosis of cancer patients, including tumor progression, metastatic seeding, and response to therapy. By using the mean combined centered expression of the CAF profiles as a proxy for cellular abundance, we set out to determine whether the cellular CAF subtypes were related to metastatic dissemination in human cohorts. We first made use of transcriptional data from a population-based nested case–control study encompassing 768 subjects. The study is designed such that 190 breast cancer patients that developed distant metastatic disease (cases) were selected from a consecutive series of individuals, and each closely matched by adjuvant therapy, age, and calendar period at diagnosis with three patients free from metastasis (controls)^28^. The gene signature for vCAFs correlated strongly to an endothelial metagene^27^ and a microvasculature signature^29^ within the dataset from the case–control study, whereas mCAFs instead were highly associated with a stroma-derived invasion signature^30^ and a stroma-related treatment-predictive signature^31^ (Fig. 5i). In strong support of the notion of CAFs as modifiers of the malignant phenotype, the vCAF gene signature was found to be an independent prognostic indicator associated with an increased risk for developing metastatic disease in both univariable and multivariable analysis in a conditional logistic regression model involving common risk factors such as lymph node status, tumor size, HER2 status, and proliferative index (Table 1). Similarly, mCAFs were also associated with risk of disseminated disease, albeit to a lesser degree (Table 1). All correlations within the case–control study were independent of the molecular subtype of the tumor (Supplementary Figure 6b,c), and both vCAFs and mCAFs were weakly anti-correlated to the PAM50 proliferation metagene^32^, ruling out general effects on cancer cell division as the link between the CAF abundance and metastatic dissemination (Fig. 5i). The observed correlations of the vCAF and the mCAF profiles to other microenvironmental gene signatures, the independence of molecular subtype, and the association to relapse were confirmed in a second clinically well-annotated gene expression dataset from 1875 patients included in the METABRIC cohort (Supplementary Figure 6d and Supplementary Table 2)^33–35^. Finally, to obtain an experimental correlate of the invasion-promoting effects of vCAFs and mCAFs suggested by our analysis, we seeded PeRo-Bas1 breast cancer cells isolated from MMTV-PyMT mice in the upper chamber of a trans-well system. The malignant cells were separated from the lower chamber, in which CAF populations isolated by FACS were seeded, by a membrane coated with Matrigel. In support of our previous findings in human tumors, both vCAFs and mCAFs significantly augmented the number of cancer cells that invaded through the matrix into the lower chamber, compared to cell culture medium alone (Fig. 5j).

**Table 1:** Univariable and multivariable conditional logistic regression models comparing patients developing metastatic disease with patients free from disseminating disease in a nested case–control study

Variable^a^	*n*	Univariate models			Multivariate models		
		HR^b^	95% CI	*P*	HR^b^	95% CI	*P*
vCAF metagene		1.47	1.23–1.76	<0.001	1.66	1.33–2.08	<0.001
mCAF metagene		1.28	1.07–1.53	0.005	1.32	1.05–1.66	0.015
Lymph node status				<0.001			0.003
Negative	304	1 (ref.)			1 (ref.)		
Positive	442	2.52	1.69–3.77		2.06	1.34–3.16	
Unknown	22	1.11	0.36–3.41		1.84	0.52–6.46	
Tumor size, mm				0.007			0.010
≤20	354	1 (ref.)			1 (ref.)		
>20	398	1.73	1.22–2.44		1.81	1.21–2.71	
Unknown	16	0.98	0.27–3.59		0.78	0.19–3.29	
Histologic grade				<0.001			0.012
Grade 1	68	1 (ref.)			1 (ref.)		
Grade 2	327	4.86	1.91–12.39		3.76	1.38–10.20	
Grade 3	328	3.94	1.52–10.23		2.51	0.87– 7.20	
Unknown	45	3.46	1.09–10.98		3.07	0.84–11.20	
HER2 status				<0.001			0.001
Negative	519	1 (ref.)			1 (ref.)		
Positive	145	2.60	1.74–3.88		2.24	1.44–3.49	
Unknown	104	0.75	0.44–1.31		0.98	0.50–1.92	
Proliferation metagene^c^		1.20	0.99–1.46	0.061	1.77	1.31–2.40	<0.001

Controls randomly matched to cases by age (<45, 45–55, 55+), adjuvant systemic therapy (endocrine therapy (ET) only, chemotherapy (CT) only, ET + CT), and calendar period of diagnosis (1997–2000, 2001–2005)

^a^Numerical variables are centered and scaled (standard deviation set to one) in the models

^b^For numerical variables, HR is the relative hazard when increasing the variable one standard deviation

^c^PAM50 proliferation index^33^, average expression of 11 proliferation genes in the PAM50 gene set

In conclusion, gene profiles of vCAFs and mCAFs were readily detectable in bulk RNA sequencing data and held biological and clinical significance for human tumors.

## Discussion

Taken together, we have significantly improved the cellular resolution of studies of CAFs by employing scRNA-seq to provide compelling evidence for the existence of at least three spatially and functionally distinct subsets of breast CAFs. Histological characterization is suggestive of distinct cellular sources of CAFs, as vCAFs, mCAFs, and dCAFs appear to originate from a perivascular location, resident fibroblasts, and malignant cells having undergone an EMT, respectively. The CAF subtypes are distinguishable within bulk transcriptional data by applying condensed gene signatures, which are conserved both between mouse and human tumors, and between distinct malignant diseases. Notably, the expression of gene signatures for different CAF subtypes, used as a proxy for the cellular abundance, held utility as independent predictors of metastatic dissemination in human breast cancer, indicating that the observed CAF subclasses have biological relevance.

Through the use of scRNA-seq and unbiased clustering of 716 individual, high-quality transcriptomes, we dissected the most prominent cellular constituent of the tumor microenvironment with high resolution, and thereby identified three distinct cellular subsets within the broadly defined CAF population. The tumor microenvironment has been suggested to harbor subpopulations of various cell types, including macrophages, endothelial cells, and CAFs, based on analysis of the expression of a limited set of markers. A recent study defined two putative subpopulations of CAFs in colorectal cancer by scRNA-seq^9^. However, the identification was based on only 17 cells denoted by the expression of only a few marker genes, making the uncertainty in classification considerable. Previous work using scRNA-seq of tumors has attempted to classify the full variety of constituent cells within cancer, thereby compromising the resolution of the studies of each individual cell type. Our approach to specifically dissect a large number of an unbiased population of CAFs using the sensitive Smart-seq2 protocol enabled enumeration of comprehensive lists of hundreds to thousands of genes that distinguished each CAF subset, thus describing the full complexity within this particular element of the tumor mass. Whether or not even deeper analysis would reveal further lower-abundance subsets of CAFs, or subdivide the clusters we observed, remains to be tested. In addition, more detailed analyses are needed in order to compare and contrast the CAF subpopulations defined by our studies with CAF subsets recently described in the literature. As an example, subpopulations of breast CAFs isolated by FACS based on differential expression of six commonly used mesenchymal cell markers were demonstrated to harbor immunosuppressive gene programs^8^. Immunomodulatory functions were not identified as a distinguishing feature of any of the CAF populations observed in our study, although more detailed analyses are warranted to investigate further as differential expression of important immune-regulatory elements was indeed detected. Furthermore, two recent studies demonstrate the promotion of cancer stem cell features by CAF subpopulations expressing CD10/Gpr77 and Hedgehog target genes such as *Fgf5*, respectively^7,10^. Intriguingly, CD10 and Gpr77 are both specifically expressed by dCAFs, raising the possibility that malignant stem cells uphold their own niche through EMT. As a mounting number of studies detailing CAF subpopulations by various technologies are presented, it will be increasingly important to develop computational tools for comparisons across platforms in order to understand the functional relationships between various subsets of CAFs.

The origin of CAFs is still contested. Conceivably, CAFs from different pedigrees may be represented within the tumor parenchyme. Indeed, our analyses suggest that the different CAF subsets may have distinct sources. Interestingly, mCAFs appear to originate from the dominating resident variety of fibroblast in the normal mouse mammary gland, based on similarities in marker expression and their peripheral location close to the surrounding normal tissue. In contrast, the malignant mammary tissue from MMTV-PyMT mice is dominated by vCAFs, possibly due to their proliferative capacity manifested in the cCAF identity. The vCAF subset shares many marker genes with pericytes, including *Cspg4*, *Rgs5*, *Pdgfrb*, and *Des*, albeit at comparably low levels. The expression of endosialin (*Tem1* or *Cd248*) was previously reported to be a marker for activated mesenchymal cells, including both CAFs and tumor pericytes^36^. Indeed, endosialin is highly expressed by vCAFs. Our observation of vCAFs being tightly vessel-associated in early-stage tumors, followed by detachment and invasion of the tumor tissue during progression, indicates a close relation between vCAFs and perivascular cells. Taken together, gene expression data and localization are conducive to speculation of a pericyte origin for vCAFs, although the concept of pericyte-to-fibroblast transition has been under recent debate^37^. The fact that vCAFs are enriched in the tumor core may indicate that hypoxia is fueling the detachment of vCAFs from its perivascular niche; a notion that is further supported by their expression of *Epas1* (HIF2-α). Finally, SDE genes denoting dCAFs were found to also be expressed by cells within the tumor epithelium, including the transgenic oncogene PyMT. Even though classical markers for EMT such as *Slug*, *Snail*, and *Twist1* were not expressed to a greater extent by dCAFs compared to other CAF populations (Supplementary Figure 7), their transcriptional signature and histological localization suggest an epithelial origin. Conceivably, our negative selection strategy may not have excluded malignant cells having undergone an EMT, since EpCAM has been reported to be downregulated during the mesenchymal transition^38^. Intriguingly, the detected dCAF SDE genes provide a unique source of new distinguishing features that aid in the functional and histological definition of EMT-cells in situ in mixed pools of non-EMT malignant cells and mesenchymal stromal cells within tissues.

CAFs are known to support many of the hallmarks of cancer^2–4^. However, recent studies of pancreatic ductal adenocarcinoma contest the view of the tumor-supportive CAF, as an increase in growth and aggressiveness was observed following eradication of α-SMA^+^ CAFs or targeting of the desmoplastic response induced by Hedgehog^39,40^. The contradictory influence of CAFs on the malignant phenotype may be explained by the existence of subpopulations of cells with opposing functions. We classified CAFs into three different subtypes based on global gene expression patterns. The functional grouping of CAFs was strongly supported by independent unsupervised clustering of the cells based on the expression of gene sets of importance for known CAF functions, such as ECM production. Additionally, mapping of cells within the tumor tissue by immunostaining of unique markers validated the spatial separation of the CAF subclasses. Of note, we observed a striking difference in the spectrum of expressed ECM genes between vCAFs, mCAFs, and dCAFs. While mCAFs abundantly produced a wide variety of matrix components, vCAFs and dCAFs were more restricted in their expression pattern, specializing in the production of basement membrane products and paracrine signaling molecules, respectively. Based on the expression of specific genes, inferences can be made as to the function of that CAF subgroup. As a case-in-point, we have recently in a separate study identified paracrine PDGF-CC signaling to CAFs as a regulator of the basal-like molecular subtype of breast cancers^41^. The receptor for PDGF-CC, i.e., PDGFRα, is exclusively expressed by mCAFs, identifying this subgroup as responsible for the specification of ERα-negativity in breast tumors. Another recent study corroborated the link between CAFs and ERα status, demonstrating that a subset of CD146^+^ CAFs increased hormone receptor expression in mammary tumors^6^. Indeed, CD146 is absent from mCAFs and exclusively expressed by vCAFs in our classification, supporting specific targeting of PDGFRα^+^/CD146^−^ CAFs as an attractive strategy to sensitize basal-like breast tumors to endocrine therapy by conversion into an ERα^+^ phenotype. Thus, apart from providing information about the functional properties of the different subclasses of CAFs, our approach also provides putative drug targets for further development.

In translational efforts, we found that the vCAF and mCAF signatures were highly conserved in patient samples of breast tumors, indicating that fibroblast subtypes representing functionally distinct biologies are a feature also of human malignancies. Cross-comparison of a range of different malignant diseases demonstrated that the CAF subpopulations that we defined were present in many, but not all, other tumor types. It is likely that the spectrum of CAF subsets within a particular tumor reflects different aspects of biology, including cell-of-origin and molecular activation status. The ability to distinguish stromal cell subclasses within data from bulk RNA-seq, opens up the possibility to use quantitative measures of microenvironmental composition as prognostic or predictive biomarkers. In support of this proposition, the signatures from vCAFs or mCAFs held prognostic capabilities by their association to metastatic dissemination in two large clinical cohorts comprising >2600 breast cancer patients. Further, the mCAF signature was correlated to a treatment-predictive stromal signature^31^. Taken together, we here present an improved cellular, molecular, and functional taxonomy of breast CAFs, opening up the possibility for development of novel targeted drugs or biomarkers of clinical significance with increased precision.

## Methods

### Cell isolation from breast tumors

All animal experiments were performed according to institutional guidelines and approved by the local ethics committee in Lund (permit number M167/15). Tumors from 14-week-old MMTV-PyMT mice were excised and the surrounding mammary fat pad removed. Tumors were minced and digested in 10 ml FACS buffer PBS, 5% Cell dissociation buffer (Gibco, 13151014), 0.2% BSA (Sigma Aldrich, 05479) containing 25 mg Collagenase II (Gibco, 1797319), 25 mg Collagenase IV (Gibco, 17104-019), 5 mg DNAse (Sigma Aldrich, DN25), for 15 min stirring at 37 °C. The digested cell suspension was strained through a 100 µm cell strainer with the plunger of a plastic syringe. After spinning down for 3 min at 300×*g*, red blood cells were lysed using RBL buffer containing 0.15 M ammonium chloride and 10 mM sodium EDTA in ddH_2_O for 30 s. Red blood cell lysis was stopped with ice-cold FACS buffer. Cells were counted after additional straining through a 70 µm mesh cells and centrifugation at 300×*g* for 3 min.

### Orthotopic transplantation models

For syngeneic models, 10^5^ 4T1 (ATCC) and 5 × 10^5^ EO771 (ATCC) tumor cells were injected in 50 µl PBS in the 4th inguinal mammary fat pad of BALB/c and C57BL/6J mice, respectively. For human xenografts, 2 × 10^6^ MDA-MB-231 cells (ATCC) were injected in 50 µl PBS in NOD.CB17-*Prkdc*^*scid*^.

### Flow cytometry and sorting

Fc regions on cells were blocked with 2 µl Fc-block (BD Pharmingen, 553141) in 50 µl FACS buffer per 10^6^ cells for 10 min on ice. For fibroblast negative selection, the cells were incubated in staining cocktail containing anti-CD31-APC (1 µl/10^6^ cells, BD Biosciences, 561814, Clone: MEC 13.3), anti-CD45-APC (1 µl/10^6^ cells, BD Biosciences, 559864, Clone: 30-F11), anti-CSPG4-AF647 (0.4 µl/10^6^ cells, Bioss, bs-4800R-A647), and anti-CD326-APC (5 µl/10^6^ cells, BD Biosciences, 563478, Clone G8.8) in FACS buffer for 30 min on ice. 4′-6′-diamidino-2-phenylindole (DAPI) was added to the cell suspension before sorting. We gated on DAPI^−^, Epcam^−^, CD31^−^, CD45^−^, NG2^−^ cells after excluding doublets. The cells were sorted using a FACSARIA II (BD Biosciences) into individual wells of 386-well plates containing lysis buffer provided by the Eukaryotic single cell facility (ESCG), SciLifeLabs, (Stockholm, Sweden). For population resorting, cells obtained from MMTV-PyMT mice crossed with the PdgfRα-EGFP reporter mouse^42^ were stained with anti-CD31-APC, anti-CD45-APC, anti-NG2-AF647, anti-CD326-APC, anti-PDGF-Rβ-biotin (Thermo Scientific, 13-1402-82) for 30 min on ice, followed by 20 min incubation on ice with streptavidin-PE/Cy7 (Thermo Scientific, 25-4317-82).

### cDNA preparation and sequencing

Lysis buffer, library preparation, and sequencing were provided by ESCG, following the Smart-Seq2 protocol^16^. Read alignment, gene-expression estimation, normalization, and quality control were provided by ESCG.

### Read alignment and estimation of gene-expression

Single-end 43 bp long reads were aligned at ESCG to mm10 mouse genome using STAR v2.3.0^43^ with default settings, and RefSeq annotation was used for gene expression quantification, which resulted in 24,490 endogenous gene counts and 92 spike-in counts, the latter was used for the analysis of technical variation.

### Normalization

Gene expression counts were normalized as reads per kilobase gene model and million mappable reads (RPKMs) using rpkmforgenes^44^.

### Data pre-processing and quality control

For data pre-processing, an estimate of systematic biases in gene expression between the 768 cells from the two plates was performed in order to exclude technical variation as a basis for any observed differences. No significant divergence in genome-wide expression pattern was found based on comparison of the total numbers of exonic reads, percentages of uniquely mapping reads, and uniquely mapping exonic reads between all cells. Furthermore, no difference in the number of highly expressed genes was detected.

To assess the quality of each cell the parameters reads, readlength, uniquely mapping reads (%), multimapping reads (%), unmapped reads (%) from the STAR log summary file were analyzed. We assessed the number of exon reads, the percentage of uniquely mapping reads, and the percentage of exon reads for each cell. In addition, we evaluated extreme RPKM values and the maximum correlation which is calculated as the maximum value of the pairwise Spearman correlations for each well. A cell was considered as an outlier if it fell beyond two standard deviations away from the mean of a QC metric distribution or in case of exon reads were below the cutoff of 10,000 reads mapped to mRNA. We excluded 52 cells that were outliers in the least two QC metrics from further analysis (Supplementary Figure 2f-j).

### Population identification

Dimensionality was reduced by *t*-SNE using the Rtsne R package with 30 initial principal components, perplexity of 27 and theta = 0.5^45^. The runs were repeated 50 times and the run with the lowest CL-divergence value containing the total costs for all objects was selected. Clusters were defined density-based spatial clustering of applications with noise (DBSCAN) clustering algorithm in R with an eps = 3.1. Two undefined outliers were manually assigned to Population 1.

### Differential expression analysis

ROTS, edgeR, DESeq2, Wilcoxon rank-sum test, and single cell differentially expressed genes (SCDE) software package were used for differential expression analysis of the defined populations. RPKM normalized data with a mean RPKM count ≥1 in all cells was used as an input except for SCDE for which raw counts were used.

### Cell cycle analysis

Cell cycle assignment was performed in R using the cyclone function^24^ included Bioconductor package “scran”^46^.

### GO-analysis

The top 150 SDE genes in each population were used as an input to investigate gene sets in the Molecular Signatures Database [http://software.broadinstitute.org/gsea/msigdb/index.jsp]^47^.

### Single cell immunofluorescence

Flow cytometry sorted bulk cells were resuspended in 100 µl PBS and spun for 2 min at 1000 rpm in a Cytospin 4 (Thermo Scientific). The cells were dried and fixed in 4% PFA for 10 min. After incubation in ice-cold acetone for 10 min and washing in PBS, all the following steps were performed in a humidified chamber. Unspecific binding sites were masked with serum-free blocking reagent (DAKO) for 90 min at room temperature. Rat-anti-PDGF-Rα (1:200, 14-1401-82, eBioscience) and anti-αSMA-Cy3 (1:100, C6198, Sigma Aldrich) were applied overnight at 4 °C. Goat-anti-rabbit-AF488 was applied 1:1000 in PBS + 1% BSA for 90 min at room temperature, followed by mounting with DAPI-containing mounting medium (Vector Laboratories). Fluorescent images were acquired with an Olympus BX63 microscope, DP80 camera, and cellSens Dimension v 1.12 software (Olympus Cooperation).

### Immunostaining

For immunofluorescence staining, tissues were preserved in 30% w/v sucrose overnight at 4 °C before embedding in OCT Cryomount (Histolab). 6 µm tissue sections were dried at room temperature and fixed in ice-cold acetone for 10 min. All the following steps were performed in a humidified chamber. After washing in PBS, sections were blocked with serum-free blocking reagent (DAKO, X090930-2), 90 min at room temperature. Primary antibodies goat-α-mouse SCRG1 (1:200, sc-165436, Santa Cruz Biotechnology), rabbit-α-mouse Nidogen-2 (1:400, ab131279, Abcam), rat-α-mouse PDGFRβ (1:200, 16-1402-82,Thermo Fisher), rat-α-mouse ki67 (1:200, 14-5698-82, Thermo Fisher), rat-α-Epcam-APC (1:200, 17-5791-82, Thermo Fisher) diluted in PBS + 1% BSA and sections were incubated overnight at 4 °C. After washing with PBS, secondary antibodies (1:1000 in PBS + 1% BSA) against the respective species were applied for 1 h at room temperature. Sections were washed and mounted in DAPI-free or DAPI-containing mounting medium.

### Immunohistochemistry

Formalin-fixed paraffin-embedded (FFPE) sections were deparaffinized for 2 h 60 °C and re-hydrated, followed by epitope retrieval in citrate buffer (pH 6) in a pressure cooker. Endogenous peroxidase activity was quenched with BLOXALL (Vector laboratories, SP-6000) for 15 min at room temperature, followed by washes with 0.05% Tween-20 in PBS. For antibodies raised in mouse, the Mouse on Mouse (M.O.M.) basic kit (Vector Laboratories, BMK-2202) was used according to the manufacturer’s datasheet, with an additional blocking step with CAS-block (Thermo Fisher, 008120) for 30 min prior to the incubation in M.O.M. diluent. Primary antibodies against Desmin (1:100, sc-23879, Santa Cruz Biotechnology) and Fibulin-1 (1:50, ab211536, Abcam) were diluted in M.O.M. diluent. CAS-block was used for the blocking (2 h) and incubation of primary antibody against PDGFR-α (1:500, D1E1E 3174, Cell Signaling Technology). Primary antibody incubation was performed overnight at 4 °C in a humidified chamber. After washing, appropriate secondary biotinylated antibodies and the ABC elite standard kit peroxidase system (Vector Laboratories) with DAB as a substrate (Vector Laboratories) were applied.

### RNA in situ hybridization

Tissues were fresh frozen in OCT Cryomount (Histolab, 45830) and 5 µm sections were stained following the RNAscope Fluorescent Multiplex Assay (Advanced Cell Diagnostics, USA) instructions. Images were acquired with a Zeiss LSM 710 laser scanning microscope.

### 2-Photon confocal microscopy

Immunostained tissues were imaged using an inverted Leica SP5-X MP multiphoton Leica microscope connected to a Ti-Sapphire laser (Spectra Physics MaiTai HP DeepSee Laser), Spectral Physics (tunable wavelength: 690 –1040 nm). The objective used was a HCX PL APO lambda blue 63 × 1.20 NA WATER UV. Tissue sections containing GFP-expressing cells were stained for expression of SCRG1 (Alexa 555) and Nidogen-2 (Alexa 647). Fibrillar collagen was imaged by means of second harmonic generation (SHG) using two-photon excitation at 892 nm and emission between 426 and 446 nm was detected using a hybrid detector (HyD SP, Leica). GFP-expressing cells were simultaneously excited using the 892 nm two-photon excitation and emitted GFP-light was collected between 505 and 550 nm using a PMT detector. SCRG1 (Alexa 555) and Nidogen-2 (Alexa 647) was excited with a supercontinuum white light laser (WLL) and emitted light (Alexa 555: 567–612 nm and Alexa 647: 650–710 nm) were detected using the hybrid detector (HyD SP, Leica). All images are from back-scattered light and captured with a resolution of 1024 × 1024 pixels, at 200 Hz.

### Transwell invasion assay

All cells were cultured at 37 °C and 5% CO_2_ and were frequently checked for mycoplasma infections using the MycoAlert^TM^ Mycoplasma Detection Kit (Lonza). 8 µm pore 24-well transwell inserts were coated with Matrigel® Growth Factor Reduced (GFR) Basement Membrane Matrix (Corning, USA) 1:3 in starvation medium (DMEM + 0.1% BSA). 10^5^ PeRo-Bas1 tumor cells (generated in-house) were seeded in the transwell insert and placed in 24-well plates containing CAFs (80% confluent) in starvation medium. Starvation medium only and FBS were used as negative and positive controls. After 24 h the transwell inserts were removed and cells on the bottom side of the membrane were fixed with 70% EtOH and stained with crystal violet. The stained area of four representative images was determined using ImageJ.

### Gene profile definition

RNA-seq data for breast cancer were downloaded from the TCGA data portal and log_2_ transformed after adding an offset of 1. For each population, the SDE genes were compacted using Spearman correlation. A gene was used as a seed and the top correlating genes to that seed were added until the average correlation between all genes was <0.7.

### Clinical samples and datasets

Human breast cancer tissue for immunostaining was provided by the Sweden Cancerome Network Breast Initiative (SCAN-B)^48^; ClinicalTrials.gov identifier NCT02306096 with approval# 2009/658 and 2009/659 by the local Ethics Review Board.

The TCGA RNAseq data were downloaded from the TCGA data portal (https://tcga-data.nci.nih.gov) 2015 on June 12th (pancreatic adenocarcinoma), September 17th (lung adenocarcinoma), and September 18th (breast carcinoma and renal clear cell carcinoma). The expression data were log_2_-transformed after addition of 1. The METABRIC data set^33,34^ was downloaded from cBioPortal for Cancer Genomics (http://www.cbioportal.org). Clinical–pathological data and intrinsic molecular subtype classifications were retrieved from the supplementary information of the publication^28^. Preprocessed microarray gene-expression data was retrieved from the European Genome-phenome Archive (EGA) with accession numbers EGAD00010000210 and EGAD00010000211. Probesets were mapped to Entrez Gene IDs using the R/Bioconductor annotation package illuminaHumanv3.db.

The nested case–control study has been described in detail previously^28,49^. In brief, women diagnosed with primary breast cancer 1997–2005 in the Stockholm health care region of Sweden were identified, and patients developing distant metastatic disease (cases) were selected and controls (free from distant disease) were randomly matched by adjuvant therapy, age, and calendar period at diagnosis. Microarray gene expression data are available at the Gene Expression Omnibus (GEO) database under accession number GSE48091. Probe sets were mapped to Entrez Gene IDs using the manufacturer’s annotations. A quality control sub-study of the nested case–control study (GEO GSE81954) was also analyzed with reassuring results (Supplementary Figure 8a,b). The nested case–control study has previously been approved for gene expression analyses by the ethics committee at Karolinska Institutet, Stockholm, Sweden.

All gene expression data analysis and statistical analysis were done in R/Bioconductor. Microarray data were log_2_ transformed, normalized probe intensity values. Expression data were collapsed to gene level using a non-specific filter keeping only the probe sets with the highest interquartile range in the case of multiple mappings to the same Entrez Gene ID. Intrinsic molecular subtypes of the tumors in the nested case–control study were determined using nearest correlations with the PAM50 centroids.

Six gene expression signatures were explored for their correlation with the CAF populations. These included endothelial/microvasculature signatures^27,29^, stroma-related signatures^27,30,31^, and a proliferation signature^32^. Gene signature scores were derived as the weighted averages of the expression values of the constituent signature genes, where the weight for each gene is +1 or −1 depending on the direction with the phenotype in the original publication. Only original genes that could be mapped to Entrez Gene IDs were used. The derived signature scores were named as EC metagene (*CDH5, CXorf36, TIE1*)^27^, Microvasculature^29^, Stroma 1^30^, Stroma 2^31^, ECM metagene (*COL1A1*, *COL1A2*, *COL3A1*)^27^, and Proliferation^32^.

In the nested case–control study, the association with risk of disseminating disease was analyzed with conditional logistic regression models. In the METABRIC study, the association with risk of breast cancer deaths was analyzed with Cox proportional hazards regression models with stratification by study site and intrinsic molecular subtype.

## Electronic supplementary material

Supplementary Information

Peer Review File

## Data Availability

ScRNA-sequencing data that support the findings of this study have been deposited in GEO with the accession code GSE111229 and will be made available upon publication of the article. Main R scripts for the analysis and necessary data are available at GitHub [www.github.com/KPLab/SCS_CAF]. A reporting summary for this article is available as a Supplementary Information file.
