# Supplementary material for: Spatially and functionally distinct subclasses of breast cancer-associated fibroblasts revealed by single cell RNA sequencing
Source: Nat Commun. 2018 Dec 4;9:5150. doi: 10.1038/s41467-018-07582-3 (PMC6279758; doi:10.1038/s41467-018-07582-3)
Supplement: Supplementary file 1 — Supplementary Information [file 41467_2018_7582_MOESM1_ESM.pdf]

Spatially and functionally distinct subclasses of breast cancer-associated fibroblasts revealed by single cell RNA sequencing

Bartoschek et al

Supplementary figure 1

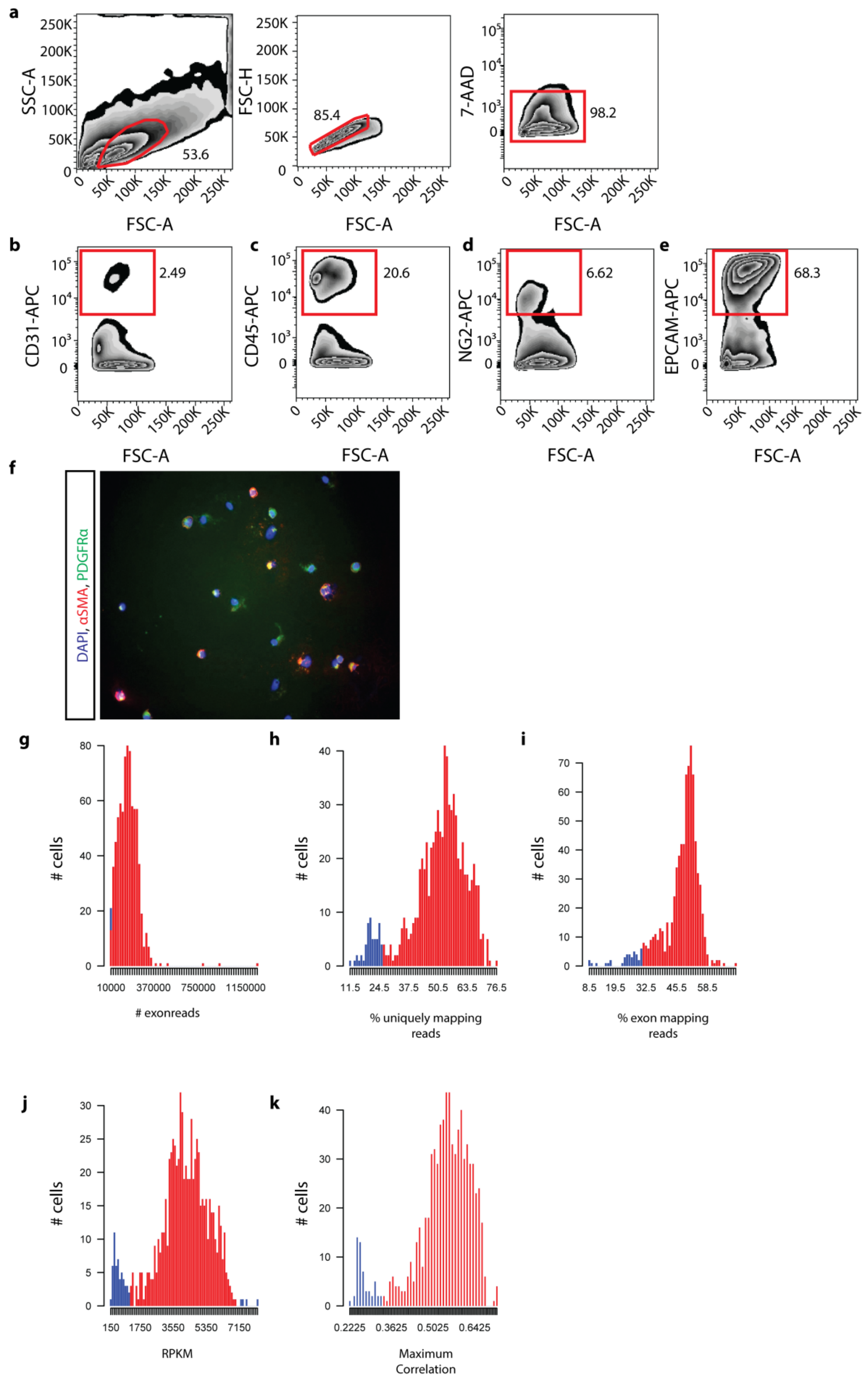

Supplementary figure 1. **Negative selection strategy and single-cell sequencing quality control.** Single staining for negative selection markers used to isolate EPCAMP^{-}PCD31P^{-}PCD45P^{-}PNG2P^{-}P fibroblasts from MMTV-PyMT tumor tissue. (**a**) Gating on cells defined as single 7-AAD- living cells. Gating on (**b**) CD31P^{+}P endothelial cells, (**c**) CD45P^{+}P blood derived cells, (**d**) NG2P^{+}P pericytes and (**e**) EPCAMP^{+}P epithelial malignant cells. (**f**) Cytospin of sorted cells, stained for CAF markers α-SMA (red) and PDGFRα (green). Quality control metrics used to exclude low quality transcriptomes (**g**) number of exon mapping reads. Cutoff: 10000 (8 cells failed). (**h**) Percentage of uniquely mapping reads. Cutoff: 26.11 % (56 cells failed). (**i**) percentage of exon mapping reads. Cutoff: 31.15% (40 cells failed). (**j**) Number of genes with reads per kilobase gene model and million mappable reads (RPKM)>1. Cutoff: 1112.76 and 7023 (56 cells failed). (**k**) Maximum correlation of each cell to all other cells. Cutoff: 0.34 (54 cells failed).

Supplementary figure 2

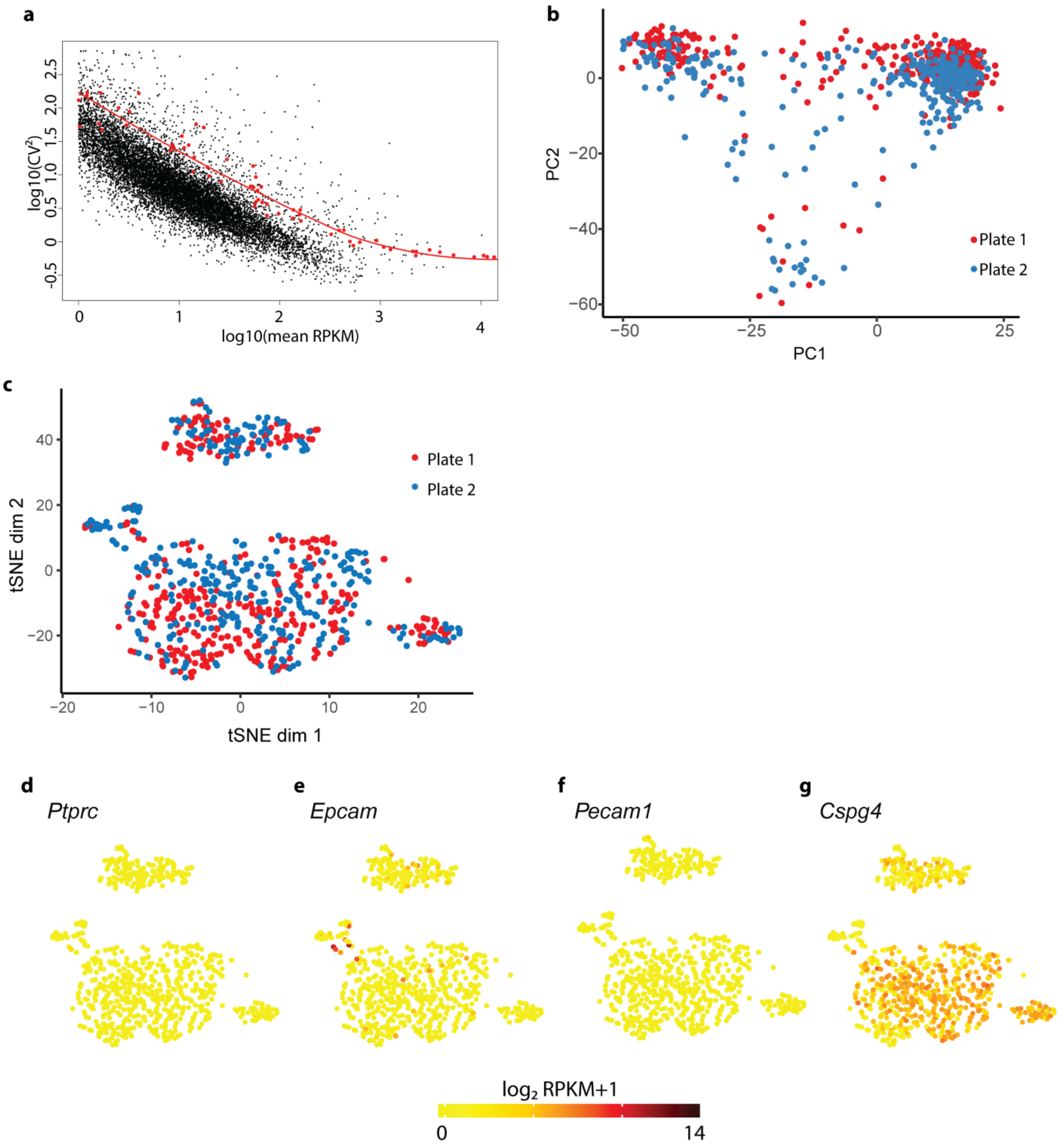

Supplementary figure 2. **Cluster description.** Variance to average detection of genes and spike-ins. (**a**) RPKM normalized gene counts (black) and spike-ins (red). (**b**) First two principal components of RPKM normalized expression data of all cells. (**c**) *t*-SNE layout colored based on the plate/tumor each transcriptome was derived. (**g-j**) Expression plot of negative selection

genes; (**g**) *Ptprc* encoding CD45, (**h**) *Epcam* (**i**) *Cspg4* encoding NG2 and (**j**) *Pecam1* encoding CD31.

Supplementary figure 3

a

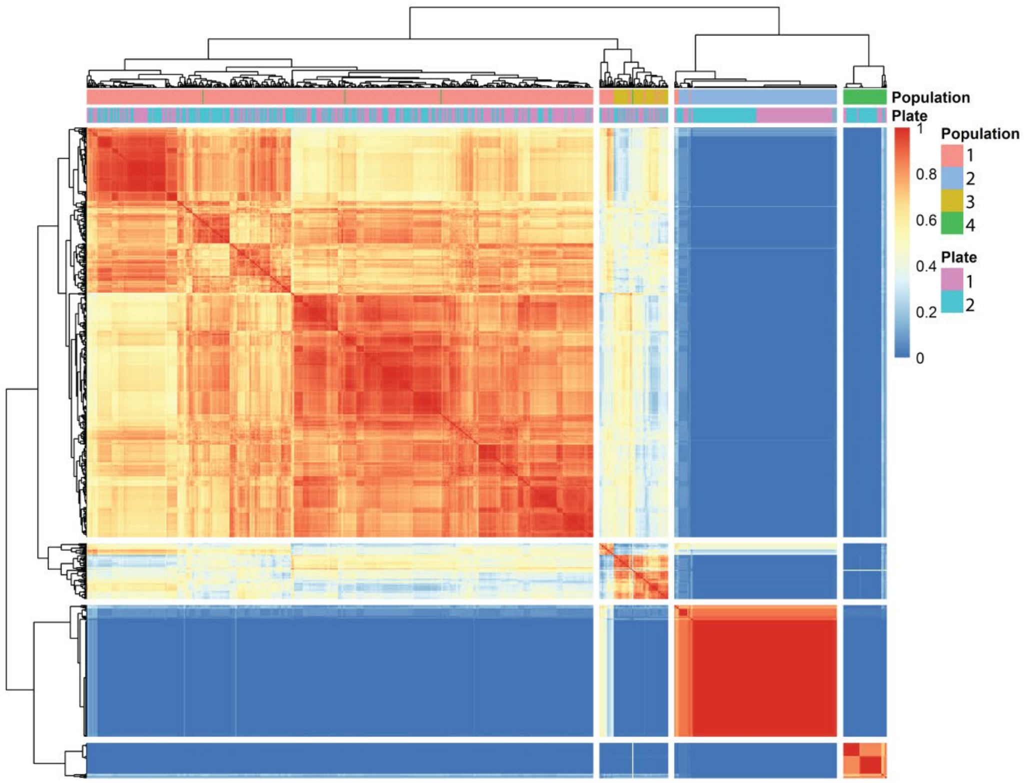

b

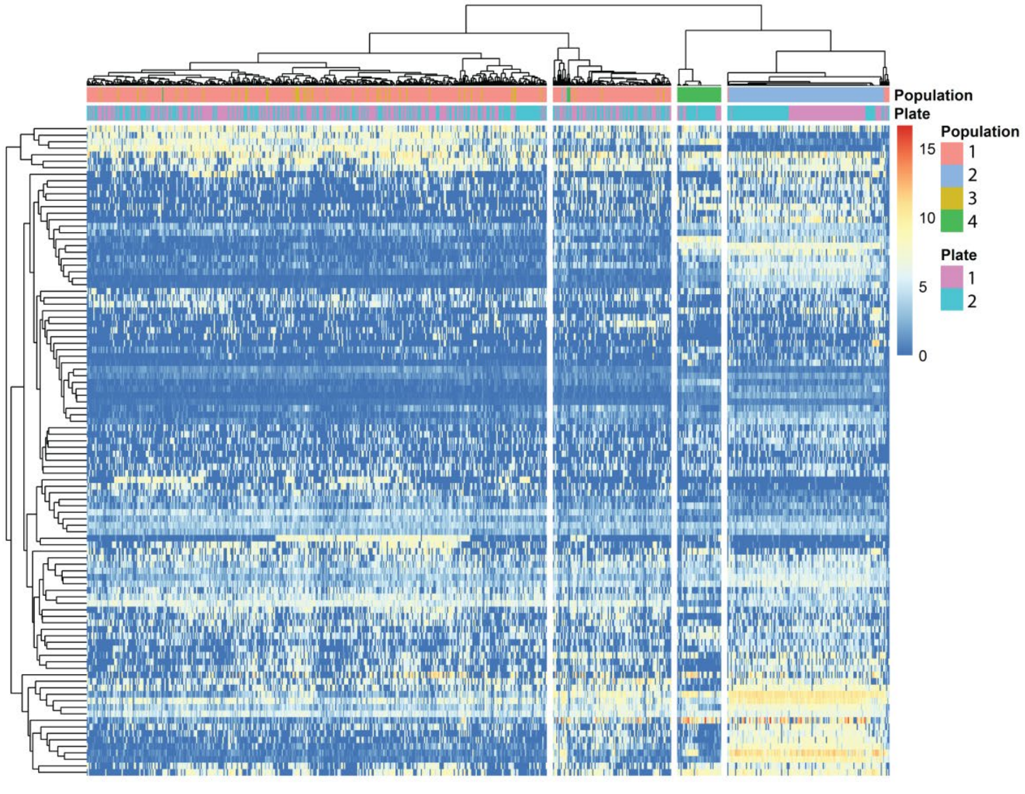

Supplementary figure 3.**Clustering using the SC3 package in R**. (**a**) Cells cluster similarly to the *t*-SNE based clustering in Figure 1D when using the entire dataset. Neglectable batch effects based on the sequencing plate can be observed in population 2. The heatmap depicts correlation between cells. (**b**) Unbiased clustering based on the expression of Matrisome (32) genes only. The clustering of populations 2 and 4 remain while populations 1 and 3 are not distinguishable based on matrisome gene expression. The heatmap depicts logR_2R(RPKM+1) gene expression.

Supplementary figure 4

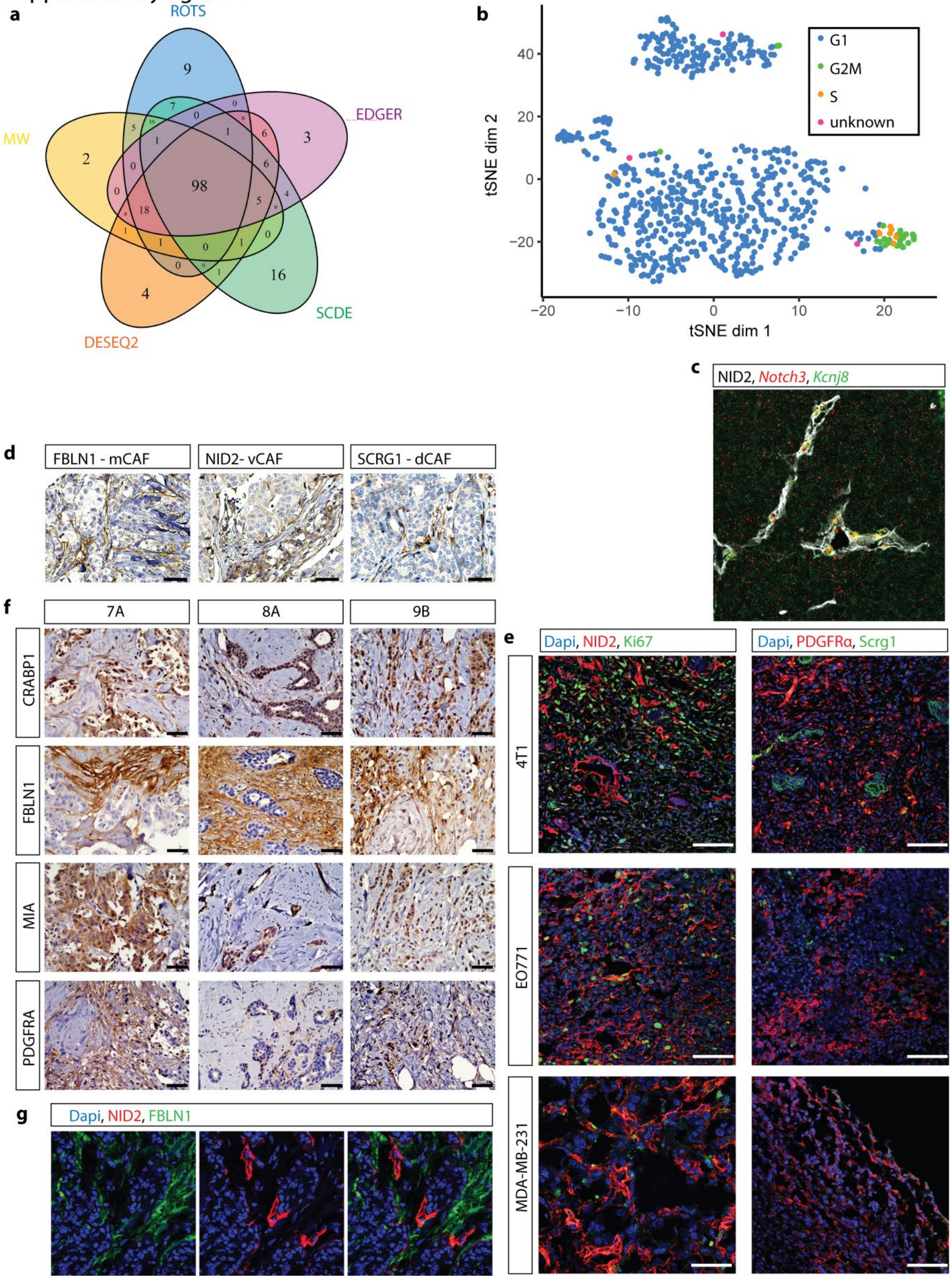

Supplementary figure 4. **Detecting differentially expressed genes in various models.** (**a**) Venn diagram of the 150 top significantly differentially expressed (SDE) genes detected in population 1 with reproducibility-optimized test statistic (ROTS), single cell differentially expressed genes (SCDE), edgeR, Wilcoxon rank-sum test (MW) and DESeq2. (**b**) Cell cycle status of cells as classified by cyclone classifier for mouse data (based on *t*-SNE layout from Figure 1D). (**c**) RNA-*in situ* hybridization for *Notch3* (red) and *Kcnj8* (green) combined with IF staining of nidogen-2. (**d**) Fibulin-1, Nidogen-2 and SCRG1 staining on human breast tumor sections obtained from The Human Protein Atlas. Scale bars 50 µm. (**e**) IF staining of breast tumors from orthotopically injected murine (4T1 and EO771) and human (MDA-MB-231) cell lines for nidogen-2 and Ki-67 (left) and PDGFRα and SCRG1 (right). (**f**) IHC staining of three human tumor samples (left to right) for FBLN1, PDGFRα (mCAF) and MIA (dCAF). Scale bar 50 µm.

Supplementary figure 5

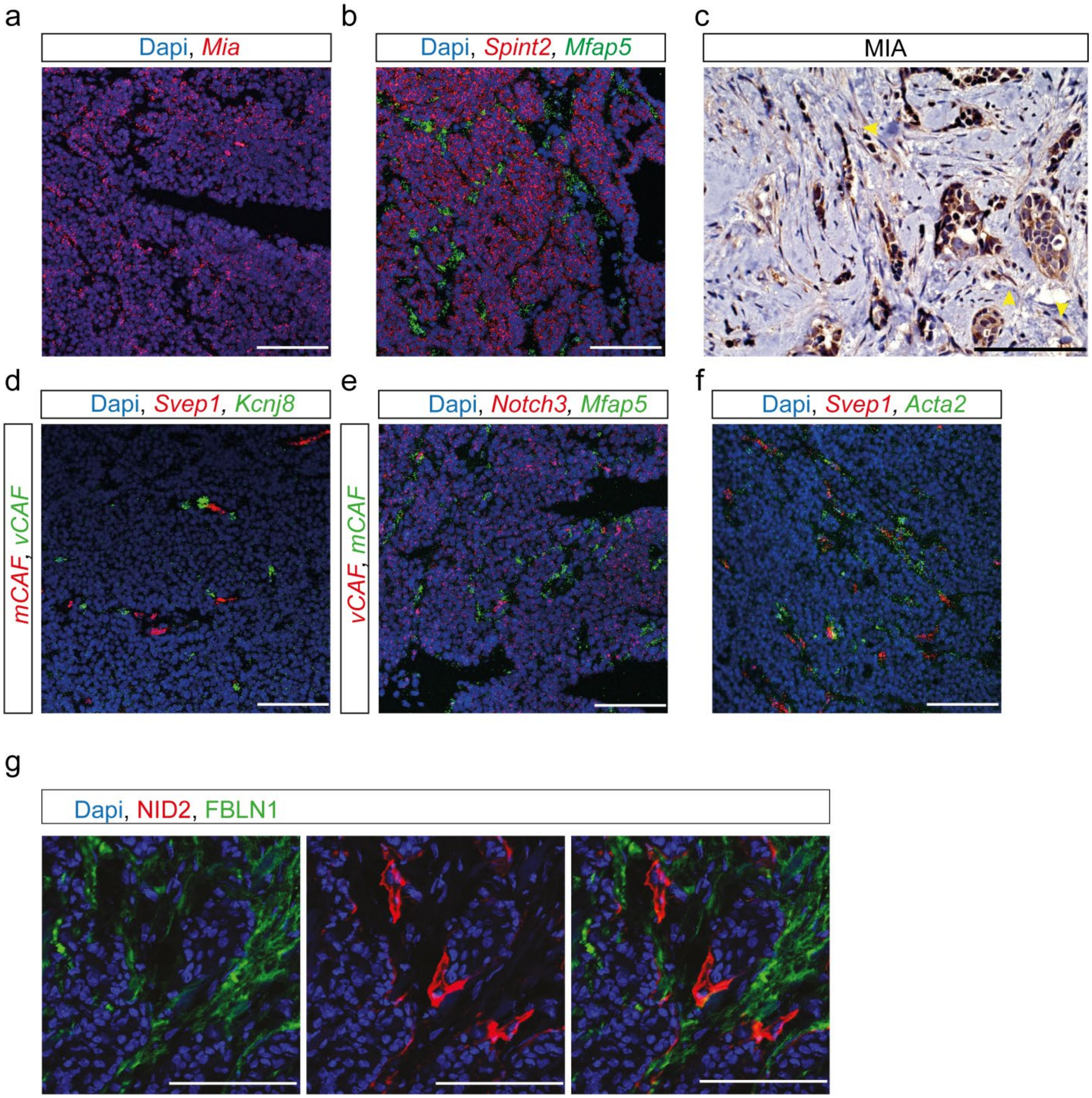

Supplementary Figure 5. Distinct expression of CAF markers. RNA *in situ* hybridization (ISH) of (**a**) Mia and (**b**) Spint2 (red) Mafap5 (green) in snap frozen MMTV-PyMT tumor sections (5 µm). Nuclei were counterstained with DAPI. (**c**) IHC of Mia in human tumor tissue sections (6 µm). Yellow arrows indicate Mia positive stromal cells. RNA-ISH of (**d**) *Svep1* and *Kcnj8*, (**e**) *Notch3* and *Mfap5* and (**f**) *Svep1* and *Acta2* on MMTV-PyMT tumor tissue sections (5 µm).

Nuclei were counterstained with DAPI (blue). Scale bars 50 μm. (**g**) IF staining of nidogen-2 (red) and fibulin-1 (green) on human breast cancer sections. Nuclei were counter stained with Dapi (blue).

Supplementary figure 6

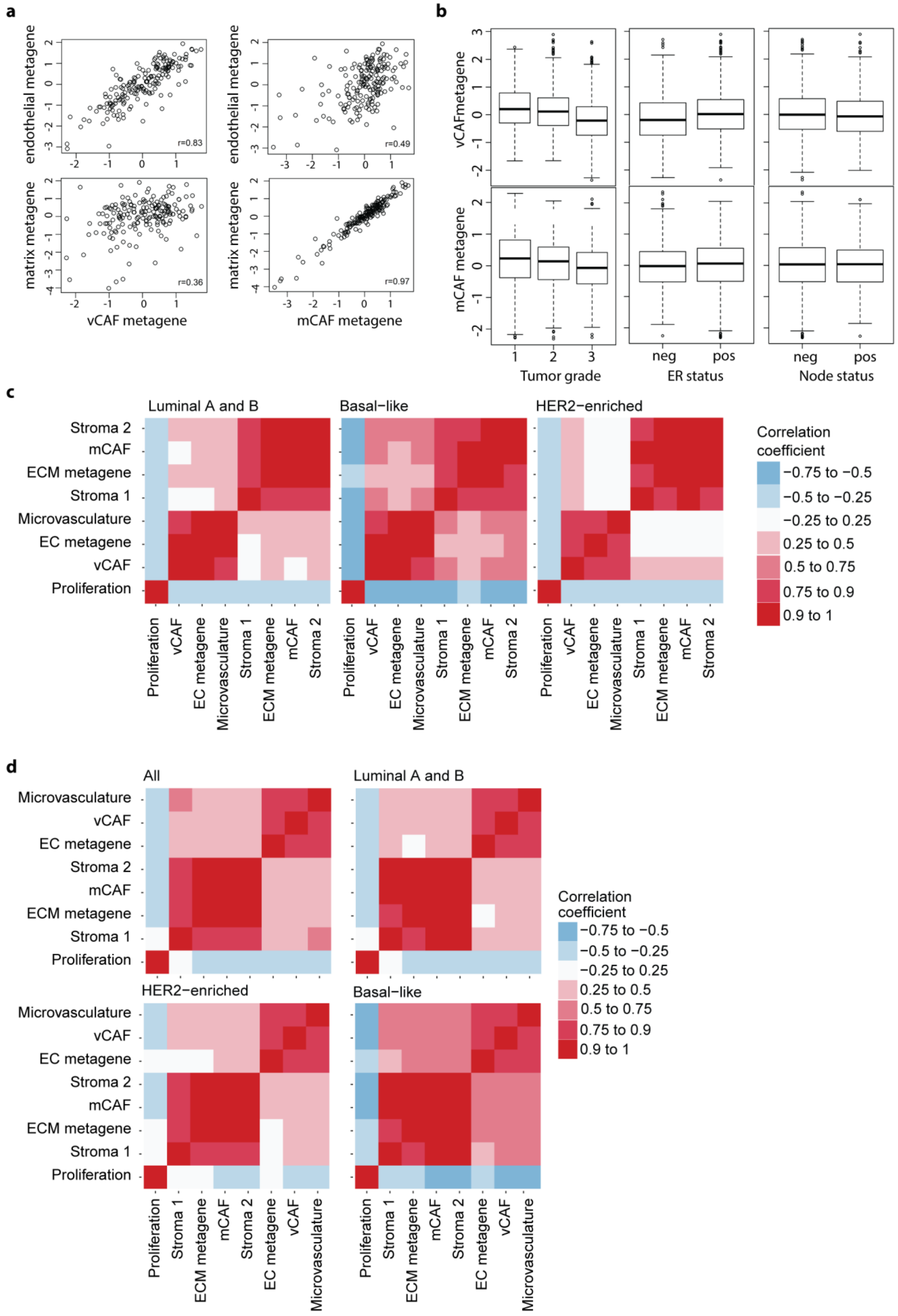

Supplementary figure 6. **Comparison of CAF gene profiles with functional metagenes in human bulk sequencing data of pancreatic ductal adenocarcinoma samples.** (**a**) Correlation of vCAF and mCAF gene profiles with an endothelial and a matrix metagene. (**b**) Tumor grade dependent expression of vCAF and mCAF gene profiles depending on tumor grade, ER status and node status. (**c**) Correlation of vCAF and mCAF profiles to functional metagenes in a nested case-control study of breast cancer depending on molecular subtype. (**d**) Correlation of vCAF and mCAF profiles to functional metagenes in the METABRIC dataset of breast cancer depending on the molecular subtype.

Supplementary figure 7

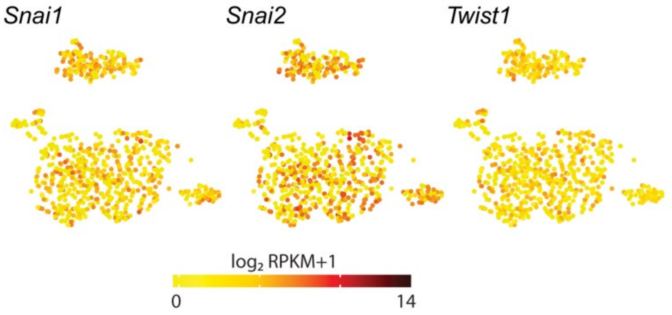

Supplementary figure 7. Feature plots of EMT genes. *Snai1*, *Snai2* and *Twist* expression is not a prominent feature of any CAF subtype.

Supplementary figure 8

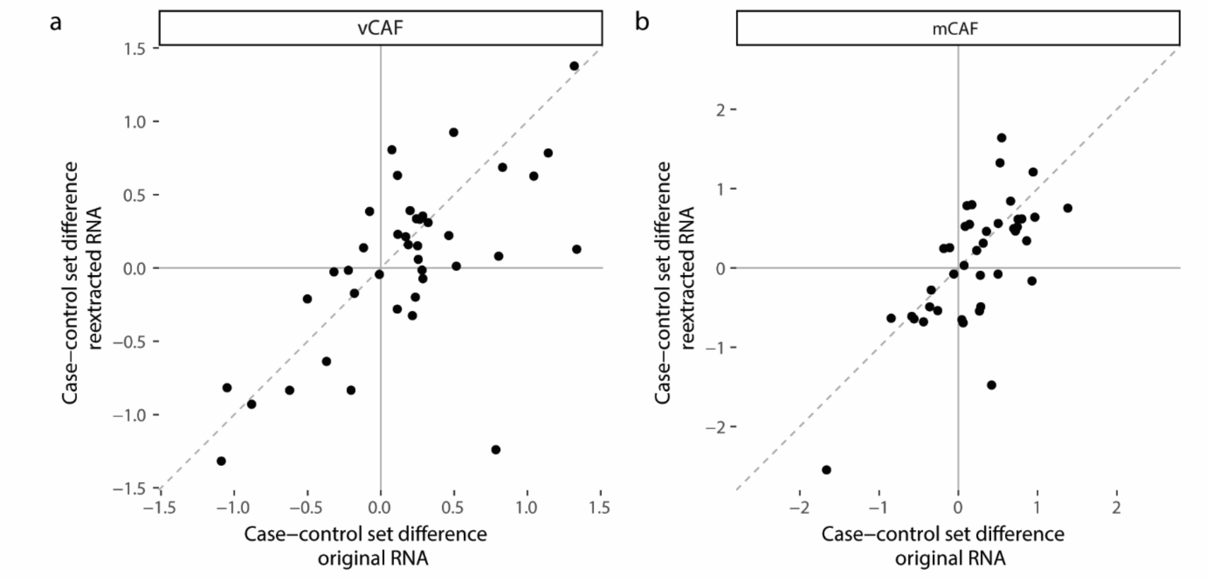

Supplementary figure 8. **Quality control sub-studies analyzing RNA from separate tumor pieces verify unbiased sampling and analysis for the vCAF gene signature** (**a**) and the mCAF gene signature (**b**) within the nested case-control study.

Supplementary Tables

Supplementary Table 1. Condensed gene profiles of CAF subpopulations based on correlation of SDE genes in human breast cancer bulk RNA-Sequencing data.

Population	Genes
vCAF	*Esam, Gng11, Higd1b, Cox4i2, Cygb, Gja4, Eng*
mCAF	*Dcn, Col12a1, Mmp2, Lum, Mrc2, Bicc1, Lrrc15, Mfap5, Col3A1, Mmp14, Spon1, Pdgfrl, Serpinf1, Lrp1, Gfpt2, Ctsk, Cdh11, Itgbl1, Col6a2, Postn, Ccdc80, Lox, Vcan, Col1a1, Fbn1, Col1a2, Pdpn, Col6a1, Fstl1, Col5a2, Aebp1*
dCAF	*Tspan2, Reck*

Supplementary table 2. Univariate and multivariate Cox regression models of breast cancer specific survival in the METABRIC study with stratification by study site and intrinsic molecular subtype. Patients with missing data on included clinical-pathological variables or intrinsic molecular subtype are excluded. * Numerical variables are centered and scaled (standard deviation set to one) in the models. † For numerical variables, HR is the relative hazard when increasing the variable one standard deviation. ‡ PAM50 proliferation index (Nielsen et al., 2010), average expression of 11 proliferation genes in the PAM50 gene set. HR, hazard ratio; ref., reference.

		Univariate models			Multivariate model A			Multivariate model B			Multivariate model C		
Variable*	n	HR†	95% CI	*P*	HR†	95% CI	*P*	HR†	95% CI	*P*	HR†	95% CI	*P*
vCAF metagene		1.11	1.00 to 1.22	.043	1.20	1.08 to 1.34	.001				1.16	1.04 to 1.30	.011
mCAF metagene		1.08	0.99 to 1.19	.094				1.16	1.04 to 1.29	.006	1.11	0.99 to 1.24	.070
Age at diagnosis, years				.137			.649			.593			.620
< 45	247	1 (ref.)			1 (ref.)			1 (ref.)			1 (ref.)		
45 to 55	364	0.74	0.54 to 1.01		0.87	0.63 to 1.19		0.85	0.62 to 1.17		0.86	0.63 to 1.19	
55+	1264	0.79	0.61 to 1.03		0.95	0.72 to 1.25		0.94	0.72 to 1.24		0.96	0.73 to 1.26	
Lymph node status				<.001			<.001			<.001			<.001
Negative	970	1 (ref.)			1 (ref.)			1 (ref.)			1 (ref.)		
Positive	905	2.67	2.20 to 3.25		2.35	1.92 to 2.87		2.37	1.94 to 2.89		2.34	1.92 to 2.86	
Tumor size, mm				<.001			<.001			<.001			<.001
≤ 20	820	1 (ref.)			1 (ref.)			1 (ref.)			1 (ref.)		
> 20	1055	1.89	1.56 to 2.31		1.56	1.27 to 1.90		1.56	1.28 to 1.91		1.56	1.27 to 1.91	
Histologic grade				.008			.614			.698			.666
Grade 1	168	1 (ref.)			1 (ref.)			1 (ref.)			1 (ref.)		
Grade 2	760	1.42	0.86 to 2.33		1.23	0.74 to 2.04		1.21	0.73 to 2.01		1.21	0.73 to 2.01	
Grade 3	947	1.84	1.11 to 3.06		1.29	0.77 to 2.16		1.25	0.74 to 2.09		1.26	0.75 to 2.12	
Proliferation metagene‡		1.35	1.17 to 1.56	<.001	1.37	1.17 to 1.60	<.001	1.33	1.14 to 1.56	<.001	1.41	1.20 to 1.66	<.001
