# Supplementary material for: Spatially and functionally distinct subclasses of breast cancer-associated fibroblasts revealed by single cell RNA sequencing
Source: Nat Commun. 2018 Dec 4;9:5150. doi: 10.1038/s41467-018-07582-3 (PMC6279758; doi:10.1038/s41467-018-07582-3)
Supplement: Supplementary file 2 — Peer Review File [file 41467_2018_7582_MOESM2_ESM.pdf]

Reviewers' comments:

Reviewer #1 (Remarks to the Author):

I reviewed this manuscript a couple of months ago for Nature Cell Biology. My major comments were as follows -

"In this manuscript, the Pietras and colleagues describe the results of single cell sequencing of 768 cancer-associated fibroblasts (CAFs) isolated from the PyMT mammary carcinoma mouse model and demonstrate 4 distinct CAF subclasses. The potential limitation of such a study is that the findings could be potentially be restricted to a single mouse model, but the authors address this by assessing how the findings relate to human breast cancers and demonstrate that the associated gene expression profiles have prognostic capability in human disease.

The results obtained will be of great interest to those working in the field, and on a more general level to those working in a variety of disease types where mesenchymal cells play a role. The key thing that these data show us, unambiguously, is that there are distinct fibroblasts subclasses associated with tumors. This is important as it impacts on our thinking about how one might go about targeting CAFs in cancer and other disease pathologies, and our thinking about CAF biology in general. Consequently, at this level, I consider this to be a very important well-conducted study that will be of value and interest to the community.

The major limitation of this study lies in the middle part of the manuscript - the authors' claims regarding the distribution and origin of the CAF subclasses (see detailed comments below).

Note: I am not an expert in the analysis of single cell sequencing data or the bioinformatics tools used in this manuscript. Consequently, I will not comment on that aspect of the presented data."

REVIEW FOR NATURE COMMUNICATION
As you can see from my NCB review comments above, I was enthusiastic about the manuscript but considered that the major limitation of this study lay in the authors' claims regarding the distribution and origin of the CAF subclasses.

The manuscript submitted to Nature Communications is essentially unchanged with regards to the data presented and no substantial additional experiments have been performed. However, the authors have included additional panels in Figures 3 and 4, which do indeed better illustrate the localization of their fibroblasts populations. The immunofluorescent images in general remain rather "dull" and it is very hard for the reader to see the different stains. I would strongly encourage the authors' to discuss with the journal using appropriate image analysis approaches to enhance these panels to ensure that the reader is able to appreciate the data as fully as possible. Bottom line, although these panels are not fully optimal, there is no question that the different CAF population markers are differentially distributed in both human breast cancers and mouse mammary gland carcinomas/xenografts.

The second point relates to the origin of the CAF subclasses. For the NCB submission, the 2nd review also bought this up and stated "However, the authors have provided inadequate evidence to support their claims that these populations of CAFS have diverse origins and functions as claimed in the title. Further the use of immunofluorescence microscopy to localize the cells is insufficient to make claims about the origin of the CAF populations. Rather lineage tracing methodology would be a more suitable approach." Personally I think that lineage tracing (which would require a very long expensive series of experiments) is beyond the scope of this manuscript. I have carefully looked that the version of the manuscript submitted to Nature Communications and I see that the authors have indeed toned down many of their statements regarding the origin/function of their CAF populations, including revising the title of the manuscript.

A minor comment. The Roswall paper is now available on PubMed i.e. the reference for this should be updated

In conclusion: The data presented in this manuscript will be of great interest to the many researchers in the CAF field. The data are also timely with the increasing interest in the community as to stromal heterogeneity and potential origins of CAFs. In my opinion there is plenty of high quality novel data here to warrant publication in Nature Communications.

Reviewer #2 (Remarks to the Author):

Bartoschek et al. report the identification of spatially and functionally distinct subclasses of breast cancer-associated CAFs by scRNAseq. In general, this is a good study which is well presented and could potentially be relevant to breast cancer patient diagnosis and prognosis. However, there are key issues that I believe the authors should address before the full potential of their findings is confirmed. I list these below:

Major points to address:

1) How reproducible are these findings?
The authors base their findings on 700 cells from two animals. The scRNAseq technology nowadays allows for more cells to be sequenced. I would like to see more cells sequenced using 10X or similar approaches.

2) On the same point – the authors use one mouse model of breast cancer. I don't think this is enough to make general statements about CAFs. The authors should also sample more mouse models of cancer (well at least one more). Even though the MMTV-PyMT is a defined model, no two tumours are the same. The authors should also sequence the tumour cells. This way they can correlate the CAF profiles to the tumour profile. The authors also need to be aware that the MMTV promoter can be leaky and express in other cell types. They might need to check the different tissues.

3) Validation of the spatial distribution of the various CAFs.
The authors use conventional IHC to demonstrate the spatial distribution of the CAF cells. These images are not convincing and do not support the claims. I think this is a key (and potentially exciting) point in the paper and I suggest that the authors should use whole tissue clearing techniques such as (Clarity) and lightsheet/confocal microscopy to visualise the different CAF cells in situ. (Lloyd-Lewis B. et al 2016 Breast cancer research) provides a good overview of how to do that on mammary tissue.

4) The authors analyse TCGA and METABRIC datasets and detect the expression signatures of aCAFs and mCAFs. They then suggest that aCAF's rather than mCAFs are invasion promoting. The authors need to provide experimental evidence to support this claim. This can be done by co-culturing the different types of CAFS with breast cancer cell lines and performing in vitro and in vivo migration assays.

5) The authors claim to have identified that mCAF, aCAF and dCAF derive from resident fibroblasts, perivasculature and tumor cells, respectively. However, all of this is mainly based on expression similarities between the CAF subtype and the proposed cell-of-origin. I doubt that this evidence is strong enough to allow conclusions like "mCAFS are derived from resident fibroblasts that are co-opted by the tumor" (p12) or "dCAFS originate from tumor cells that underwent EMT" (p13). Especially the notion that tumor cells undergo EMT to produce their own CAFs is quite a big (and exciting) statement. Yet, this statement requires more evidence than provided by the authors. There are numerous examples where the expression profile of a cell does not provide any information about its cellular origin. In my opinion, these statements can only be made if the authors had some sort of information on the lineage of these cells from e.g. genetic marking as in lineage tracing experiments. The authors need to either loosen the statements or perform additional experiments to support these claims.

Comments on the scRNA-seq analysis:
1. The method section about the analysis is too brief and needs more detail. In addition, all the code should be made available by the authors, preferentially on github or elsewhere.

2. P6.L18 "In order to prove the robustness of the data […]", Using different normalization methods does not prove the robustness of your data. In general the use of two different normalization methods (RPKM and deconvolution) is confusing to the reader, and it is not always clear which is used at which step, and why. Further, it is stated that the "differences in size factors was negligible", if that's the case, then the parallel use of both methods is redundant. In relation to this, there seems to be some issue with the computation of HVGs, it's unexpected that 9027 genes are defined as highly variable, this is essentially the majority of the identified genes. It is also not clear how the fit in S2b was computed, assumingly that was only done on spike-ins? To circumvent this the authors could either define the top 10% of genes with the highest biological variance as HVG or fit the technical trend to all endogenous genes, which might give a better estimate.

3. The method section states that DBSCAN was used for clustering, which is not mentioned once in the main text. Instead the authors say that "tSNE grouped the cells distinctly into four clusters". tSNE

is merely a visualization tool for high-dimensional data and does not allow any inference on clustering. The authors should be more specific aobut what was actually used to derive the labelling of Pop1-4.

4. I struggled to follow the approach of the clustering on the "matrisome" geneset (p8). It seems convoluted to cluster the data again, just to show that the previously defined clusters differ in their expression of genes related to ECM. That should be easy to show by normal differential expression analysis of the clusters.

5. P9 "In line with the previous clustering, each cellular subset was validated by the exclusive expression of the top SDE genes". This is a circular argument, the SDE genes were defined by DE analysis using the clusters as covariates and can thus not be an independent validation of the clustering. This should be rephrased.

Minor Comments:
1. The authors should integrate their findings better into the current standard of knowledge, in the introduction as well as the discussion. E.g. Costa et al. ("Fibroblast Heterogeneity and Immunosuppressive Environment in Human Breast Cancer", Cancer Cell 2018) identified 4 subclasses of CAFs in BC, how do they relate to the clusters identified here? Also, there are other datasets with scRNA-seq profiles of CAFs from patients (e.g. https://www.biorxiv.org/content/biorxiv/early/2017/11/08/215954.f ull.pdf), would be useful to look for similarities differences?)

2. p2.l4 the word "unbiased" might be misleading as there is marker based cell sorting involved. Even if it is a negative selection strategy.

3. P4.l15 "each CAF subset was clearly discriminated functionally", the authors don't perform any functional studies in this manuscript, and this claim is therefore not fully supported.

4. P10 In the section of "aCAFS are predominatly present in the tumor core and originate from a perivascular location." It should be noted that the markers used will also label the cCAF cluster.

5. P12 " Based on being the dominant CAF subclass in the normal mammary gland", CAFs in the normal mammary gland??!

6. P20 "data not shown" why not?

7. Supplementary table 2 legend – references a paper from the year 2020!

Reviewer #1 (Remarks to the Author):
I reviewed this manuscript a couple of months ago for Nature Cell Biology. My major comments were as follows -

"In this manuscript, the Pietras and colleagues describe the results of single cell sequencing of 768 cancer-associated fibroblasts (CAFs) isolated from the PyMT mammary carcinoma mouse model and demonstrate 4 distinct CAF subclasses. The potential limitation of such a study is that the findings could be potentially be restricted to a single mouse model, but the authors address this by assessing how the findings relate to human breast cancers and demonstrate that the associated gene expression profiles have prognostic capability in human disease.

The results obtained will be of great interest to those working in the field, and on a more general level to those working in a variety of disease types where mesenchymal cells play a role. The key thing that these data show us, unambiguously, is that there are distinct fibroblasts subclasses associated with tumors. This is important as it impacts on our thinking about how one might go about targeting CAFs in cancer and other disease pathologies, and our thinking about CAF biology in general. Consequently, at this level, I consider this to be a very important well-conducted study that will be of value and interest to the community.

The major limitation of this study lies in the middle part of the manuscript - the authors' claims regarding the distribution and origin of the CAF subclasses (see detailed comments below).

Note: I am not an expert in the analysis of single cell sequencing data or the bioinformatics tools used in this manuscript. Consequently, I will not comment on that aspect of the presented data."

REVIEW FOR NATURE COMMUNICATION
As you can see from my NCB review comments above, I was enthusiastic about the manuscript but considered that the major limitation of this study lay in the authors' claims regarding the distribution and origin of the CAF subclasses.

Authors:
Please note that we have changed the nomenclature of the observed CAF subsets such that the major CAF subset originating in juxtaposition with the vasculature is now denoted vascular CAFs (vCAFs).

The manuscript submitted to Nature Communications is essentially unchanged with regards to the data presented and no substantial additional experiments have been performed. However, the authors have included additional panels in Figures 3 and 4, which do indeed better illustrate the localization of their fibroblasts populations. The immunofluorescent images in general remain rather "dull" and it is very hard for the reader to see the different stains. I would strongly encourage the authors' to discuss with the journal using appropriate image analysis approaches to enhance these panels to ensure that the reader is able to appreciate the data as fully as possible. Bottom line, although these panels are not fully optimal, there is no question that the different CAF population markers are differentially distributed in both human breast cancers and mouse mammary gland carcinomas/xenografts.

Authors:
We have enhanced the quality and color representation of all images from immunostaining of tissues. In addition, we have performed 2-photon confocal imaging to provide even more conspicuous evidence for distinct spatial distribution of the observed CAF populations and their

relationship to other compartments of the tumor tissue. In support of our previous observations, we now report distinct localization of mCAFs within the collagen-rich fibrotic streaks of the tumor tissue, as well as dCAF marker localization close to, or within, the malignant epithelium. These results are now presented in Fig. 4e and described on page 12 in the revised manuscript.

The second point relates to the origin of the CAF subclasses. For the NCB submission, the 2nd review also bought this up and stated "However, the authors have provided inadequate evidence to support their claims that these populations of CAFS have diverse origins and functions as claimed in the title. Further the use of immunofluorescence microscopy to localize the cells is insufficient to make claims about the origin of the CAF populations. Rather lineage tracing methodology would be a more suitable approach." Personally I think that lineage tracing (which would require a very long expensive series of experiments) is beyond the scope of this manuscript. I have carefully looked that the version of the manuscript submitted to Nature Communications and I see that the authors have indeed toned down many of their statements regarding the origin/function of their CAF populations, including revising the title of the manuscript.

Authors:
We thank the referee for this comment and are happy that the revisions we have previously made are satisfactory. We have again reviewed all our statements about the origin of CAF subsets to ascertain that we are not overstating the conclusions drawn from the data.

A minor comment. The Roswall paper is now available on PubMed i.e. the reference for this should be updated

Authors:
The updated reference has now been included, thank you for pointing out this oversight.

Reviewer #2 (Remarks to the Author):
Bartoschek et al. report the identification of spatially and functionally distinct subclasses of breast cancer-associated CAFs by scRNAseq. In general, this is a good study which is well presented and could potentially be relevant to breast cancer patient diagnosis and prognosis. However, there are key issues that I believe the authors should address before the full potential of their findings is confirmed.

Authors:
Please note that we have changed the nomenclature of the observed CAF subsets such that the major CAF subset originating in juxtaposition with the vasculature is now denoted vascular CAFs (vCAFs).

I list these below:

Major points to address:
1) How reproducible are these findings?
The authors base their findings on 700 cells from two animals. The scRNAseq technology nowadays allows for more cells to be sequenced. I would like to see more cells sequenced using 10X or similar approaches.

Authors:
In the current study, we have run RNA sequencing of 768 CAFs according to the Smart-seq2 protocol (Picelli et al, Nature Methods 10:1096-1098, 2013; Picelli et al, Nature Protocols 9:171.181, 2014). The Smart-seq2 protocol allows for generation of a full-length cDNA library that can be used for high coverage deep sequencing from both 3'- and 5'-ends, resulting in both high accuracy and high sensitivity of the data (see quality controls depicted in Fig. 1c and SFig. 1g-k). The high quality of the data, however, makes the cost of analyzing cells in the thousands prohibitory. In contrast, while being considerably cheaper per cell analyzed, the single cell RNA sequencing provided by the 10X Genomics system results in significantly shorter and fewer reads per cell, reducing the sensitivity and accuracy. The 10X Genomics system is thus very suitable to accurately portray cellular subsets in unselected isolations of cells. Our strategy, however, included an enrichment for a broadly defined cell type through negative selection. Thus, we opted to use the Smart-seq2 protocol due to the need to be able to reproducibly, accurately and with high sensitivity distinguish between cell populations that may be defined by subtle differences in the expression of genes with low absolute expression. We do not exclude that analysis of a higher number of CAFs would reveal additional CAF subsets, but we estimate that these would in such cases represent fewer than 1% of the total pool. Nevertheless, the analysis of our data from Smart-seq2 sequencing resulted in robust identification of 4 CAF subsets that were subsequently verified by immunostaining and analysis of bulk RNAseq data, demonstrating the high accuracy and sensitivity of our methodology and the validity of our approach.

2) On the same point – the authors use one mouse model of breast cancer. I don't think this is enough to make general statements about CAFs. The authors should also sample more mouse models of cancer (well at least one more). Even though the MMTV-PyMT is a defined model, no two tumours are the same. The authors should also sequence the tumour cells. This way they can correlate the CAF profiles to the tumour profile. The authors also need to be aware that the MMTV promoter can be leaky and express in other cell types. They might need to check the different tissues.

Authors:
We thank the referee for this important comment. However, using immunostaining we have already assessed the localization and spatial separation of CAF subtypes in additional mammary carcinoma syngrafts (4T1 and EO771), xenografts (MDA-MB-231), as well as in human breast cancers (see Fig. 3d, g-h, SFig 4d-f and 5c and g). The conclusion after validation in additional tumor types remains that specific markers for each CAF subset are expressed by distinct populations of cells with a mesenchymal morphology residing in the tumor stroma. Nevertheless, we agree that for future studies it will be important to understand how prevalent the observed subclasses of CAFs are in a variety of malignant diseases, as we did observe differences in the ratio of CAF populations.

Concerning the comment by the referee about correlating CAF profiles to tumor profiles, we agree that it would be highly warranted to investigate differences in stromal make-up depending on oncogenic drivers, mutational status, molecular subtype etc, but this is outside the scope of the current investigation.

Finally, we have analyzed the patency of the MMTV promoter by immunostaining sections of lungs, livers, and mammary fat pads (both female and male) for PyMT protein without finding evidence for ectopic expression outside of the malignant mammary epithelium (see Fig. 1 for Editorial review).

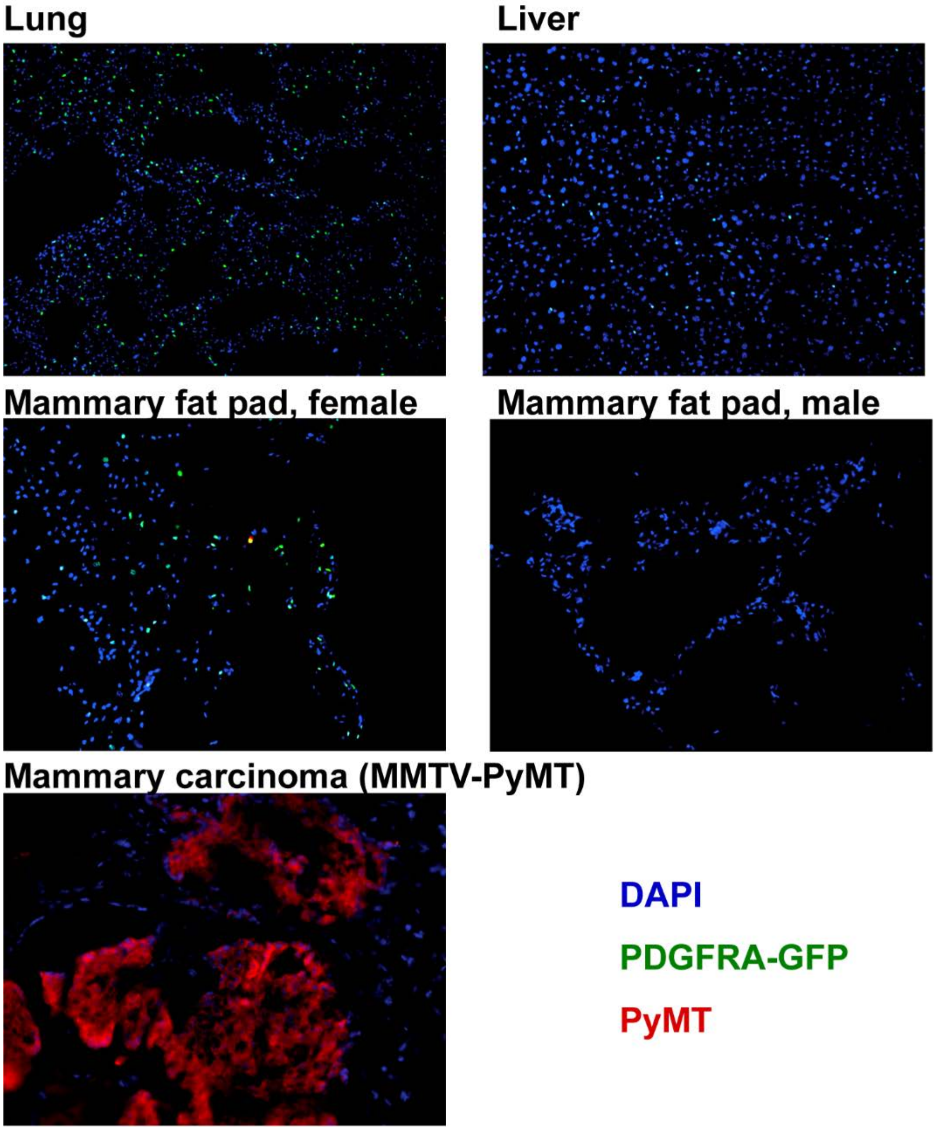

Figure 1 for Editorial review. Immunostaining of various tissues from double transgenic MMTV-PyMT;PDGFRα–GFP mice for the PyMT protein. No ectopic expression of PyMT was observed outside of the tumor parenchyma. Please note that the areas imaged of the mammary fat pad were chosen not to contain tumor or ductal structures to demonstrate that the surrounding tissue does not ectopically express the transgenic oncogene.

3) Validation of the spatial distribution of the various CAFs.
The authors use conventional IHC to demonstrate the spatial distribution of the CAF cells. These images are not convincing and do not support the claims. I think this is a key (and potentially exciting) point in the paper and I suggest that the authors should use whole tissue clearing techniques such as (Clarity) and lightsheet/confocal microscopy to visualise the different CAF cells in situ. (Lloyd-Lewis B. et al 2016 Breast cancer research) provides a good overview of how to do that on mammary tissue.

Authors:
To improve the quality of the images depicting CAF subsets *in situ* in tumor tissue, we have now performed 2-photon confocal microscopy. The enhanced image analysis further supports the distinct spatial localization of the CAF subsets. Moreover, the images illustrate: 1) mCAFs embedded in collagen-rich streaks, thus supporting their role as producers of ECM, 2) vCAFs juxtaposed with the nidogen-containing vascular basement membrane, and 3) dCAFs seemingly emanating from the malignant epithelium. The new data is now included in Fig. 4e and discussed on page 12 of the revised manuscript.

4) The authors analyse TCGA and METABRIC datasets and detect the expression signatures of aCAFs and mCAFs. They then suggest that aCAF's rather than mCAFs are invasion promoting. The authors need to provide experimental evidence to support this claim. This can be done by co-culturing the different types of CAFS with breast cancer cell lines and performing in vitro and in vivo migration assays.

Authors:
The multivariable analysis of gene expression data from two well-characterized cohorts of human breast cancer specimens revealed that both vCAFs and mCAFs were associated with an increased risk of metastatic dissemination (see Table 1 and STable 2). To provide experimental support for this claim, we isolated vCAFs and mCAFs by FACS using specific cell surface markers. Next, the CAF populations were each seeded in the lower well of a trans-well system in which the chambers were separated by a Matrigel-coated membrane. Quantification of the number of PeRo-Bas1 tumor cells derived from a tumor of the MMTV-PyMT mouse model that invaded from the upper chamber to the lower chamber indeed revealed that both vCAFs and mCAFs significantly augmented invasion of malignant cells compared to cell culture medium alone. These data are now included in Fig. 5j and discussed on page 15 in the revised manuscript.

5) The authors claim to have identified that mCAF, aCAF and dCAF derive from resident fibroblasts, perivasculature and tumor cells, respectively. However, all of this is mainly based on expression similarities between the CAF subtype and the proposed cell-of-origin. I doubt that this evidence is strong enough to allow conclusions like "mCAFS are derived from resident fibroblasts that are co-opted by the tumor" (p12) or "dCAFS originate from tumor cells that underwent EMT" (p13). Especially the notion that tumor cells undergo EMT to produce their own CAFs is quite a big (and exciting) statement. Yet, this statement requires more evidence than provided by the authors. There are numerous examples where the expression profile of a cell does not provide any information about its cellular origin. In my opinion, these statements can only be made if the authors had some sort of information on the lineage of these cells from e.g. genetic marking as in lineage tracing experiments. The authors need to either loosen the statements or perform additional experiments to support these claims.

Authors:
We agree that our conclusions concerning the origin of the different CAF subsets would be strengthened by lineage tracing experiments. Nevertheless, we also agree with Referee 1 and the Editor that such experiments are outside of the scope for the current study and would not be without their own caveats. We suggest that vCAFs, mCAFs and dCAFs are derived from peri-vascular cells, resident fibroblasts and malignant cells based on several lines of evidence. Firstly, in early stages of tumor development, vCAFs are exclusively found in a peri-vascular location (Fig. 3c). During tumor progression, vCAFs are dislocated from the vasculature and subsequently constitute a large part of the cancer stroma (Fig. 3c). Secondly, based on flow cytometry analysis of the non-transgenic mouse mammary fat pad, almost 90% of fibroblasts in the tissue express signature markers of mCAFs

(Fig. 3i-j). As the tumor progresses, the proportion of mCAFs within the total CAF pool decreases, but the spatial localization of mCAFs in fibrotic streaks remains (Fig. 3c). Thirdly, we base the proposition of dCAFs as malignant cells in disguise through EMT on the fact that dCAFs express the transgenic oncogene PyMT that is under the control of a promoter only active in mammary epithelium, as well as on the fact that dCAFs localize within the malignant epithelium and share specific markers with a subset of cancer cells on the epithelial/stromal border (Fig. 3b and e). In the revised manuscript, we have scrutinized all our statements about the origin of CAF subsets in order not to overstate our conclusions.

Comments on the scRNA-seq analysis:
1. The method section about the analysis is too brief and needs more detail. In addition, all the code should be made available by the authors, preferentially on github or elsewhere.

Authors:
We have now included more details about our analysis in the methods section, and a detailed record of all our procedures will be deposited to GitHub.

2. P6.L18 "In order to prove the robustness of the data […]", Using different normalization methods does not prove the robustness of your data. In general the use of two different normalization methods (RPKM and deconvolution) is confusing to the reader, and it is not always clear which is used at which step, and why. Further, it is stated that the "differences in size factors was negligible", if that's the case, then the parallel use of both methods is redundant. In relation to this, there seems to be some issue with the computation of HVGs, it's unexpected that 9027 genes are defined as highly variable, this is essentially the majority of the identified genes. It is also not clear how the fit in S2b was computed, assumingly that was only done on spike-ins? To circumvent this the authors could either define the top 10% of genes with the highest biological variance as HVG or fit the technical trend to all endogenous genes, which might give a better estimate.

Authors:
We agree that the use of both normalization methods can be confusing to the reader. To simplify and improve clarity we now only used RPKM-based normalization in the revised manuscript. The amount of highly variable genes will only influence the initial dimensionality reduction by *t*-SNE and PCA, as the differential gene expression analysis was done with all genes with an average gene expression (RPKM) equal or higher than one.

3. The method section states that DBSCAN was used for clustering, which is not mentioned once in the main text. Instead the authors say that "tSNE grouped the cells distinctly into four clusters". tSNE is merely a visualization tool for high-dimensional data and does not allow any inference on clustering. The authors should be more specific aobut what was actually used to derive the labelling of Pop1-4.

Authors:
DBSCAN was now added to the results section of the revised manuscript. DBSCAN was used to label cells according to their position on the *t*-SNE plot. We are aware of the potential drawbacks of this procedure and therefore verified the population assignment by hierarchical clustering and single-cell toolkits such as the Seurat toolkit, which is widely used in the field (e.g. https://www.nature.com/articles/s41467-018-05220-6), however still remains a black-box in many situations. With DBSCAN and the other approaches (including SC3), we obtained highly similar results, indicating both a robustness of the data, as well as validity to use DBSCAN. We would be

happy to provide graphical representations to back up this approach, but have chosen not to include it here or in the revised manuscript for the sake of clarity.

4. I struggled to follow the approach of the clustering on the "matrisome" geneset (p8). It seems convoluted to cluster the data again, just to show that the previously defined clusters differ in their expression of genes related to ECM. That should be easy to show by normal differential expression analysis of the clusters.

Authors:
The referee is correct in that these data do not add extra information as compared to the differential expression analysis, but it does give a conveniently defined gene set for the major functional contribution of fibroblasts, i.e. matrix production. The aim of the clustering based on Matrisome data was both to show that CAF subclasses express different sets of ECM related genes and to show that in the absence of confounding cell cycle genes, cCAFs cluster together with vCAFs, indicating their shared origin.

5. P9 "In line with the previous clustering, each cellular subset was validated by the exclusive expression of the top SDE genes". This is a circular argument, the SDE genes were defined by DE analysis using the clusters as covariates and can thus not be an independent validation of the clustering. This should be rephrased.

Authors:
We agree that this statement was misleading. We have rephrased the sentence in the revised manuscript, which now simply states that differentially expressed genes are represented in the heatmap (see page 7 in the revised manuscript).

Minor Comments:
1. The authors should integrate their findings better into the current standard of knowledge, in the introduction as well as the discussion. E.g. Costa et al. ("Fibroblast Heterogeneity and Immunosuppressive Environment in Human Breast Cancer", Cancer Cell 2018) identified 4 subclasses of CAFs in BC, how do they relate to the clusters identified here? Also, there are other datasets with scRNA-seq profiles of CAFs from patients (e.g. https://www.biorxiv.org/content/biorxiv/early/2017/11/08/215954.full.pdf), would be useful to look for similarities differences?)

Authors:
We agree with the referee and have improved the integration of our findings into the current standard of knowledge, see page 4 of the Introduction and page 17 of the Discussion of the revised version of the manuscript.

2. p2.l4 the word "unbiased" might be misleading as there is marker based cell sorting involved. Even if it is a negative selection strategy.

Authors:
The phrasing has been changed to avoid confusion and the word “unbiased” has been removed.

3. P4.l15 "each CAF subset was clearly discriminated functionally", the authors don't perform any functional studies in this manuscript, and this claim is therefore not fully supported.

Authors:
The sentence has been revised and now reads "…each CAF subset was clearly discriminated by the expression of gene sets related to different functions…".

4. P10 In the section of "aCAFS are predominatly present in the tumor core and originate from a perivascular location." It should be noted that the markers used will also label the cCAF cluster.

Authors:
The sentence has been amended accordingly and now only speaks of "cells expressing vCAF markers".

5. P12 " Based on being the dominant CAF subclass in the normal mammary gland", CAFs in the normal mammary gland??!

Authors:
The sentence has been amended accordingly.

6. P20 "data not shown" why not?

Authors:
These data have now been included as SFig. 7 in the revised manuscript.

7. Supplementary table 2 legend – references a paper from the year 2020!

Authors:
The reference has been amended.

REVIEWERS' COMMENTS:

Reviewer #2 (Remarks to the Author):

The authors have addressed my comments. I am happy to support publication.
